# Functional pH-Responsive Nanoparticles for Immune Reprogramming in MSS Colorectal Cancer via ER Stress-Induced Proteostasis Disruption, PD-L1-Targeting miRNA, and TLR7 Activation

**DOI:** 10.3390/pharmaceutics17111503

**Published:** 2025-11-20

**Authors:** Yu-Li Lo, Hua-Ching Lin, Ching-Yao Li, Bryant Huang, Ching-Ping Yang, Hui-Yen Chuang, Tsui-Fen Chou

**Affiliations:** 1 Department and Institute of Pharmacology, National Yang Ming Chiao Tung University, Taipei 112, Taiwan; 2 Faculty of Pharmacy, National Yang Ming Chiao Tung University, Taipei 112, Taiwan; 3 Research Fellow, Taipei Veterans General Hospital, Taipei 112, Taiwan; 4 Division of Colorectal Surgery, Cheng-Hsin General Hospital, Taipei 112, Taiwan; 5 Department of Healthcare Information and Management, Ming Chuan University, Taoyuan 320, Taiwan; 6 Department of Medical Laboratory Science and Biotechnology, Central Taiwan University of Science and Technology, Taichung 412, Taiwan; 7 Department of Biomedical Imaging and Radiological Sciences, National Yang Ming Chiao Tung University, Taipei 112, Taiwan; 8 Division of Biology and Biological Engineering, California Institute of Technology, Pasadena, CA 91125, USA; tfchou@caltech.edu; 9 Proteome Exploration Laboratory, Beckman Institute, California Institute of Technology, Pasadena, CA 91125, USA

**Keywords:** colorectal cancer, solid lipid nanoparticles, endoplasmic reticulum stress, immune checkpoint modulation, tumor microenvironment

## Abstract

**Background:** Colorectal cancer (CRC), particularly the microsatellite-stable (MSS) subtype, remains largely unresponsive to immune checkpoint inhibitors (ICIs) due to immune escape, tumor-associated macrophage (TAM) enrichment, and cytokine-driven suppression that sustain a TAM-dominant tumor microenvironment (TME). To overcome these barriers, a pH-responsive solid lipid nanoparticle (SLN) system was engineered to co-deliver CB-5083 (a VCP/p97 inhibitor), miR-142 (a PD-L1-targeting microRNA), and imiquimod (R, a TLR7 agonist) for spatially confined induction of endoplasmic reticulum stress (ERS) and immune reprogramming in MSS CRC. **Methods:** The SLNs were coated with PEG–PGA for pH-triggered de-shielding and functionalized with PD-L1- and EGFR-binding peptides plus an ER-homing peptide, enabling tumor-selective and subcellular targeting. **Results:** The nanoplatform displayed acid-triggered PEG–PGA detachment, selective CRC/TAM uptake, and ER localization. CB-mediated VCP inhibition activated IRE1α/XBP1s/LC3II, PERK/eIF2α/ATF4/CHOP, and JNK/Beclin signaling, driving apoptosis and autophagy, while miR-142 suppressed PD-L1 expression and epithelial–mesenchymal transition markers. R facilitated dendritic cell maturation and M1 polarization. Combined CB + miR + R/SLN-CSW suppressed IL-17, G-CSF, and CXCL1, increased infiltration of CD4^+^ and CD8^+^ T cells, reduced Tregs and M2-TAMs, and inhibited tumor growth in CT-26 bearing mice. The treatment induced immunogenic cell death, reprogramming the TME into a T cell-permissive state and conferring resistance to tumor rechallenge. Biodistribution analysis confirmed tumor-preferential accumulation with minimal off-target exposure, and biosafety profiling demonstrated low systemic toxicity. **Conclusions:** This TME-responsive nanoplatform therefore integrates ERS induction, checkpoint modulation, and cytokine suppression to overcome immune exclusion in MSS CRC, representing a clinically translatable strategy for chemo-immunotherapy in immune-refractory tumors.

## 1. Introduction

Colorectal cancer (CRC) remains a leading cause of cancer-related mortality worldwide, with microsatellite-stable (MSS) tumors representing the majority of cases and showing poor responsiveness to current immunotherapies [1]. CT26, a widely used murine CRC model, exhibits an MSS, mismatch repair-proficient phenotype, making it suitable for preclinical studies of immunotherapy-resistant CRC [2]. Unlike microsatellite instability–high (MSI-H) tumors, MSS CRC exhibits an immunologically ‘cold’ tumor microenvironment (TME), characterized by impaired cytotoxic T lymphocyte (CTL) recruitment, accumulation of immunosuppressive tumor-associated macrophages (TAMs), and persistent secretion of cytokines and chemokines such as interleukin (IL)-17, granulocyte colony-stimulating factor (G-CSF), and IL-8 (CXCL1) [1,3]. These features establish a TAM-dominated, immune-excluded niche that is largely refractory to immune checkpoint inhibition, highlighting a critical unmet need for strategies that can both activate antitumor immunity and remodel suppressive TMEs [4].

Addressing this therapeutic challenge requires strategies that simultaneously activate antitumor immunity and reprogram suppressive TAM populations. In this context, valosin-containing protein (VCP/p97) inhibition with CB-5083 (CB) disrupts proteostasis and induces endoplasmic reticulum (ER) stress (ERS), triggering immunogenic cell death (ICD) hallmarked by calreticulin exposure, HMGB1 release, and ATP secretion [5]. Yet, systemic VCP inhibition may compromise antigen presentation and T cell priming [6], highlighting the need for combinatorial approaches. miR-142, a PD-L1-targeting immunomodulatory microRNA, restores T-cell cytotoxicity, promotes M1-like macrophage polarization via HMGB1 modulation, and restrains epithelial–mesenchymal transition (EMT) through TGF-β signaling, thereby enhancing both innate and adaptive immunity [5,7]. Imiquimod (R837; R), a Toll-like receptor 7 (TLR7) agonist, further promotes dendritic cell (DC) maturation and Th1-skewed T cell activation, although systemic administration is limited by cytokine-mediated toxicity [8,9].

To achieve tumor-specific delivery and coordinated activation of these pathways, we designed a multifunctional solid lipid nanoparticle (SLN) platform functionalized with PD-L1- and EGFR-targeting peptides, an ER-homing sequence, and a pH-labile poly(glutamic acid)–poly(ethylene glycol) (PGA–PEG) coating [5,7].

This architecture enables systemic stability, pH-triggered exposure of targeting ligands, and ER-localized payload release in the acidic TME [5,7,10]. By co-encapsulating CB, miR-142, and R837, this system aims to remodel the immunologically cold MSS CRC TME, reinstate antitumor immunity, and support novel combinatorial immunotherapy approaches in the CT26 murine model. Importantly, this design differs from prior SLN-based pancreatic cancer studies by integrating CRC-specific immunological considerations and TME reprogramming, emphasizing novelty and translational relevance.

**Scheme 1:**
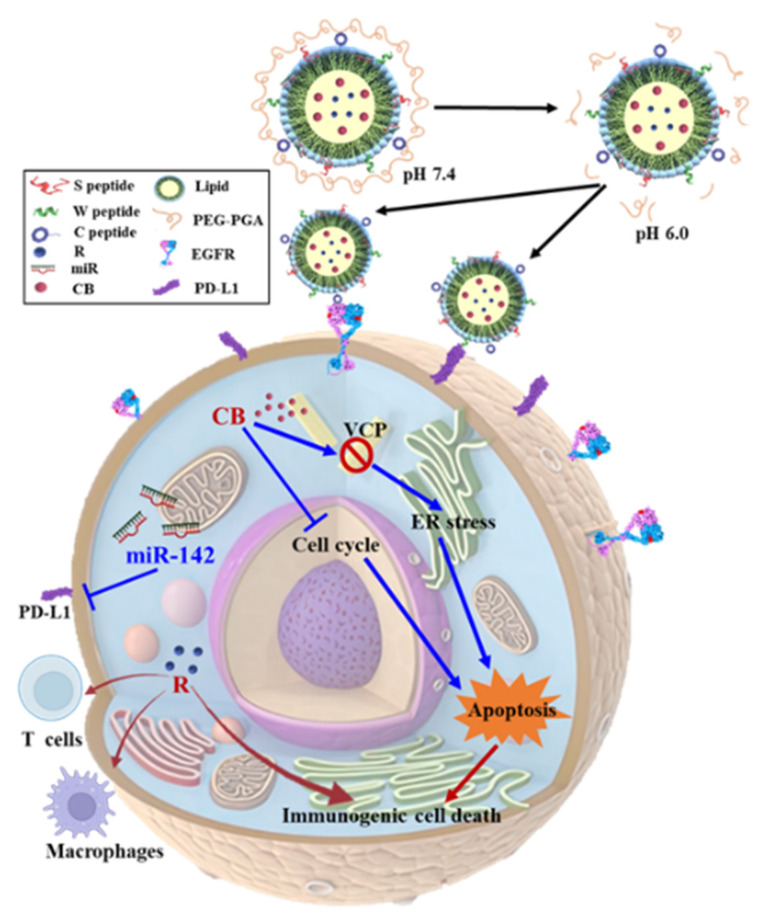
Integrated nanoplatform design for proteostasis-targeted immunotherapy in microsatellite-stable colorectal cancer. A multi-functional solid lipid nanocarrier system was engineered to co-deliver the VCP/p97 inhibitor CB-5083, miR-142, and the TLR7 agonist R837 (imiquimod) for inducing endoplasmic reticulum stress-driven immunogenic cell death in CRC. The platform incorporates hierarchical targeting through surface-conjugated peptides: C-peptide for EGFR recognition on tumor cells, W-peptide for PD-L1-expressing tumor-associated macrophages, and S-peptide for ER-specific intracellular trafficking. A stimulus-responsive polyglutamic acid-polyethylene glycol (PGA-PEG) corona shields the nanocarrier during systemic circulation at physiological pH (7.4), then undergoes acid-triggered dissociation within the tumor microenvironment (pH~6.0) through carboxyl group protonation, enabling ligand exposure and payload release at the target site.

To evaluate the therapeutic potential of this multifunctional SLN system, we systematically assessed its physicochemical properties, pH-responsive ligand exposure, and ER-targeted payload release. In vitro studies focused on proteostasis disruption, ER stress induction, and ICD in CT26 cells, while in vivo experiments examined TAM reprogramming, T-cell infiltration, and cytokine modulation. Collectively, these studies were designed to determine whether simultaneous proteostasis disruption, PD-L1 inhibition, and TLR7 activation could reprogram the TAM-dominated, immunosuppressive TME into an immune-permissive environment, providing mechanistic and translational insights for combinatorial immunotherapy in MSS CRC (Scheme 1).

## 2. Materials and Methods

### 2.1. Materials

Poly(L-glutamic acid) (PGA) was obtained from Vedan Biotechnology Corporation (Taichung, Taiwan). DOTAP and DSPE-PEG derivatives were sourced from Avanti Polar Lipids (Alabaster, AL, USA). The TLR7 agonist R837 (R) and CB were purchased from MedChemExpress (Monmouth Junction, NJ, USA). FAM-labeled and unlabeled miR-142 oligonucleotides were custom synthesized by GenePharma (Shanghai, China). The targeting peptides were prepared by Kelowna International Scientific (Taipei, Taiwan). Cholesterol, Tween^®^ 80, and paraformaldehyde were acquired from Acros Organics (Geel, Antwerp, Belgium). Cell culture media and supplements were provided by Gibco (Carlsbad, CA, USA). Additional analytical-grade reagents were procured from Cayman Chemical (Ann Arbor, MI, USA), Merck KGaA (Darmstadt, Germany), and MilliporeSigma (Burlington, MA, USA).

### 2.2. Synthesis of pH-Sensitive PGA-PEG

The pH-sensitive polymer PGA-PEG was synthesized by coupling PGA (Vedan Biotechnology Corporation, 100 mg, molecular weight 15–50 kDa) with methoxy PEG amine (mPEG-NH_2_; Nanocs, New York, NY, USA, molecular weight 2000 Da, 50 mg) using N-hydroxysuccinimide (NHS; Alfa Aesar, Ward Hill, MA, USA, 11.5 mg, 0.1 mmol) and 1-ethyl-3-(3-dimethylaminopropyl) carbodiimide hydrochloride (EDC; Acros Organics, 19.2 mg, 0.1 mmol) at a molar ratio of 1:1:1:1 in 10 mL of aqueous buffer (pH 7.4, 50 mM phosphate buffer). The mixture was stirred continuously using a magnetic stirrer (300 rpm) at 25 °C overnight under nitrogen atmosphere to prevent oxidation. Following dialysis (MWCO 3.5 kDa, Spectrum Laboratories, Rancho Dominguez, CA, USA) against phosphate-buffered saline (PBS, pH 7.4, 4 × 1 L, changed every 6 h) for 48 h at 4 °C, the product was freeze-dried using a lyophilizer at −50 °C and <0.1 mbar for 48 h. Successful conjugation of PGA-PEG was confirmed by proton ^1^H nuclear magnetic resonance (^1^H NMR; 400 MHz, Bruker Avance III, Billerica, MA, USA) in deuterium oxide (D_2_O). This acid-labile polymer was designed to remain shielded in circulation but de-shield in the acidic MSS CRC tumor microenvironment (pH~6.0–6.5), enabling ligand exposure and payload release through protonation of carboxyl groups (-COOH) on PGA at acidic pH, leading to reduced electrostatic repulsion and polymer detachment.

### 2.3. Synthesis of Lipid–Peptide Conjugates

Lipid-peptide conjugates (DSPE-PEG-C, -S, or -W) were synthesized as described previously [7] with minor modifications. Briefly, DSPE-PEG-maleimide was reacted with each peptide (C-, S-, or W-peptide) in a 1:1 molar ratio overnight (16–18 h) in aqueous buffer at 25 °C under gentle magnetic stirring (200 rpm) protected from light. The maleimide group of DSPE-PEG reacted with the thiol group of cysteine residues in the peptides via Michael addition, forming stable thioether linkages. The conjugation product was dialyzed against PBS (MWCO 3.5 kDa, changed every 8 h) for 24 h at 4 °C and freeze-dried as described above. Successful conjugation was confirmed by matrix-assisted laser desorption/ionization time-of-flight (MALDI-TOF) mass spectrometry (Bruker, Billerica, MA, USA) in positive ion mode using α-cyano-4-hydroxycinnamic acid (MedChemExpress, Monmouth Junction, NJ, USA) as the matrix.

### 2.4. Preparation of CB + miR + R/SLN–CSW and pH-Responsive CB + miR + R/PGA–SLN–CSW

The SLNs were prepared using a modified emulsification-solvent evaporation method combined with electrostatic adsorption. Briefly, monostearin (glyceryl monostearate), cholesterol, and DOTAP were dissolved in methanol to form a lipid phase at 65 °C under magnetic stirring (400 rpm). Subsequently, CB, R, and DSPE-PEG-peptide conjugates were added sequentially to the lipid phase and stirred for 30 min at 65 °C to ensure complete dissolution and molecular mixing, followed by the addition of Tween^®^ 80 (0.1% *w*/*v*) and mixing for 1 h. The anionic miR was incorporated by dropwise addition via a micropipette into the pre-formed SLN dispersion under gentle stirring to allow electrostatic complexation. For the preparation of pH-responsive CB + miR + R/PGA-SLN-CSW, the pH-sensitive polymer PGA-PEG (0.1% *w*/*v* in PBS pH 7.4) was added dropwise to the pre-formed SLN suspension (containing CB, miR, R, and peptide conjugates) and mixed at 25 °C for 2 h under gentle magnetic stirring to allow polymer adsorption onto the nanoparticle surface.

### 2.5. Physicochemical Characterization and Long-Term Stability

The particle size, polydispersity index (PDI), and zeta potential of the nanoparticles were measured by dynamic light scattering (DLS) and laser Doppler electrophoresis using a Zetasizer Nano ZS (Malvern Instruments, Worcestershire, UK) equipped with a 633 nm He-Ne laser at 25 °C and a detection angle of 173° (backscattering mode). Each sample was diluted 1:20 in PBS (pH 7.4, filtered through 0.22 μm membrane) and equilibrated for 2 min before measurement, then analyzed in triplicate (*n* = 3) with 12 sub-runs per measurement. The results were expressed as mean ± standard deviation. The refractive index was set to 1.47 for lipids and 1.33 for PBS. Size distribution was analyzed using the intensity-weighted distribution (Z-average) and reported as mean diameter ± SD. To determine the EE% and DL% of CB- or miR-loaded nanoparticles, 1 mL of nanoparticle suspension was ultracentrifuged at 15,000 rpm for 60 min at 4 °C using a Beckman Coulter ultracentrifuge. The supernatant (containing free unencapsulated drug) was carefully removed without disturbing the pellet, and the resulting pellets were washed once with cold PBS (500 μL), re-centrifuged under the same conditions, and then lysed in 0.5% Triton X-100 (500 μL, incubated at 37 °C for 30 min with vortexing every 10 min to ensure complete lysis of nanoparticles). Following centrifugation at 15,000 rpm for 30 min at 4 °C to pellet debris, the concentrations of CB and miR in both the supernatant (free drug) and the lysed pellet (encapsulated drug) were quantified using a UV/Vis spectrophotometer (Ultrospec 8000, Biochrom, Holliston, MA, USA) at wavelengths of 280 nm for CB and a NanoDrop spectrophotometer (Thermo Fisher Scientific, Waltham, MA, USA) at 260 nm for miR, respectively. The EE% and DL% were calculated according to standard equations.

EE% = [(W_e_ − W_f_)/W_e_] × 100% (1)

DL% = [(W_e_ − W_f_)/W_t_] × 100% (2)

where W_e_ represents the initial weight of CB or miR, W_f_ denotes the weight of CB or miR in the filtrate, and W_t_ corresponds to the total nanoparticle weight.

The morphological features of the nanoparticles were examined by transmission electron microscopy (TEM; JEM-1400 PLUS, JEOL, Tokyo, Japan). For TEM imaging, nanoparticle suspensions (10 μL, diluted 1:100 in deionized water) were deposited on carbon-coated copper grids (300 mesh, Electron Microscopy Sciences, Hatfield, PA, USA), fixed by adding 2% glutaraldehyde (5 μL) for 5 min, and then negatively stained with 2% uranyl acetate (10 μL for 1 min). Multiple fields (at least 10 random fields per sample) were examined, and representative images were captured. To evaluate long-term stability, particle size, PDI, and zeta potential were monitored at predetermined time points over a 24-week period under controlled storage conditions (4 °C in amber glass vials, protected from light, *n* = 3 independent batches). At each time point, samples were brought to room temperature, gently mixed by inversion (10 times), and analyzed immediately. Physical stability was defined as a <10% change in particle size and <0.05 increase in PDI.

### 2.6. pH-Responsive Characteristics

To investigate the acid-sensitive behavior and confirm the de-shielding mechanism under mildly acidic conditions, particle size and zeta potential of CB- and/or miR-loaded formulations were measured at pH 7.4 and 6.0 using a Zetasizer Nano-ZS (Malvern Instruments).

### 2.7. Cell Lines and Culture

Murine colorectal carcinoma CT-26 cells (MSS phenotype, derived from BALB/c mice, ATCC catalog CRL-2638), RAW 264.7 murine macrophage (derived from BALB/c mice, ATCC catalog TIB-71), and IEC-6 rat intestinal epithelial cells (non-transformed normal intestinal epithelial cells, ATCC catalog CRL-1592) were obtained from the Bioresource Collection and Research Center (Hsinchu, Taiwan). CT-26 and IEC-6 cells were cultured in RPMI-1640 medium, while RAW 264.7 cells were cultured in DMEM, all supplemented with 10% fetal bovine serum (FBS; Gibco) and 1% penicillin–streptomycin–glutamine (PSG; Corning, NY, USA). The CT-26 line was explicitly chosen to model the poor T cell infiltration and TAM-rich environment characteristic of MSS CRC.

### 2.8. Intracellular Uptake and Trafficking Studies

Confocal laser scanning microscopy (CLSM) imaging was used to detect PD-L1/EGFR dual-targeting and ER localization in the MSS CRC context. CB was fluorescently labeled with 1,1′-dioctadecyl-3,3,3′,3′-tetramethyl indocarbocyanine perchlorate (DiI) for visualization. For trafficking, CT-26 cells were treated for 1, 3, and 24 h, stained for nuclei and ER (CytoPainter ER Staining Kit; Abcam, Cambridge, UK), and immunolabeled for EGFR and PD-L1. Briefly, cells were fixed with 4% paraformaldehyde for 15 min, permeabilized with 0.1% Triton X-100 in PBS for 10 min, blocked with 5% bovine serum albumin (BSA) in PBS for 1 h at room temperature, and incubated overnight at 4 °C with primary antibodies: rabbit anti-EGFR (1:200) and mouse anti-PD-L1 (1:200). After washing three times with PBS-Tween 20 (0.05%), cells were incubated with Alexa Fluor 488-conjugated goat anti-rabbit IgG (1:500) and Alexa Fluor 647-conjugated goat anti-mouse IgG (1:500) for 1 h at room temperature in the dark. Imaging was performed with an Olympus CLSM (FV10i, Tokyo, Japan). For the internalization of DiI-CB and FAM-miR, CT-26 and IEC-6 cells were incubated with DiI-CB (200 nM), FAM-miR (100 nM), and/or R (1.5 µM) formulations for 24 h. Uptake was quantified by flow cytometry (FACSCalibur, BD Biosciences, San Jose, CA, USA) using 488 nm (FAM detection, FL1 channel) and 561 nm (DiI detection, FL2 channel) lasers. A minimum of 10,000 gated events (based on forward scatter and side scatter to exclude debris) were acquired per sample. Data were analyzed using FlowJo v10 software (FlowJo LLC, Ashland, OR, USA).

### 2.9. Assessment of Cytotoxicity and Synergistic Effects by Sulforhodamine B (SRB) Assay

CT-26 and IEC-6 cells were treated with different miR-, CB-, and/or R-loaded formulations. Cells were then fixed with 1% trichloroacetic acid (TCA) at 4 °C for 1 h. Fixed cells were stained with 50 μL per well of 0.04% SRB (sulforhodamine B, Sigma-Aldrich, Saint Louis, MO, USA) dissolved in 1% acetic acid for 30 min at room temperature with gentle shaking, and washed with 1% acetic acid. After air drying, 10 mM Tris base was added to each well, and absorbance at 540 nm was measured using an ELISA reader (TECAN, Männedorf, Switzerland). To assess synergistic effects, CT-26 cells were treated with varying concentrations of miR and/or CB. The combination index (CI) was analyzed using CompuSyn software, v. 1.0 (Paramus, NJ, USA) from SRB-derived dose–response data following the Chou-Talalay method. CI values were interpreted as follows: CI < 0.7 (strong synergism), 0.7–0.85 (moderate synergism), 0.85–0.9 (slight synergism), 0.9–1.1 (additive effect), and >1.1 (antagonism). All experiments were performed in triplicate.

### 2.10. Assessment of Reactive Oxygen Species (ROS)

Following a 24 h treatment with various formulations (CB: 200 nM; miR: 100 nM; R: 1.5 μM) or vehicle control, CT-26 cells seeded at 5 × 10^4^ cells per well in 8-well chambered coverslips were washed twice with PBS and stained with 4′,6-diamidino-2-phenylindole (DAPI; Millipore Sigma, Burlington, MA, USA) for nuclear counterstaining and 2′,7′-dichlorodihydrofluorescein diacetate (DCFH-DA; Millipore Sigma) for ROS detection (20 min incubation for both stains). Cells were fixed with 4% paraformaldehyde for 10 min, and images were subsequently acquired using CLSM (Olympus FV10i). For quantitative analysis, intracellular DCF fluorescence was then measured via flow cytometry (FACSCalibur).

### 2.11. Apoptosis and Cell Cycle Analysis

To quantify apoptotic and necrotic cell populations, an Annexin V-fluorescein isothiocyanate (FITC)/propidium iodide (PI) apoptosis detection kit (Strong Biotech Corporation, Taipei, Taiwan) was utilized after 24 h of treatment. Cell suspension (~1 × 10^5^ cells) was transferred to flow cytometry tubes and incubated with Annexin V-FITC and PI (50 μg/mL) for 15 min at room temperature in the dark. Samples were analyzed within 1 h. Fluorescence was measured using FL1 (FITC, 530 nm) and FL3 (PI, 670 nm) channels, acquiring 10,000 events per sample. Cell populations were classified as follows: viable (Annexin V^−^/PI^−^), early apoptotic (Annexin V^+^/PI^−^), late apoptotic (Annexin V^+^/PI^+^), and necrotic (Annexin V^−^/PI^+^). Data were analyzed using FlowJo v10 software with appropriate compensation settings. The results were expressed as percentage of cells in each quadrant. For cell cycle analysis, CT-26 cells were seeded at 3 × 10^5^ cells per well in 6-well plates, cultured overnight, and treated for 24 h as described above. Cells were then fixed with 70% ice-cold ethanol at −20 °C overnight and subsequently stained with PI (50 μg/mL), 0.1% Triton X-100, and RNase A (Millipore Sigma) for 30 min at 37 °C in the dark. Cell cycle distribution across G0/G1, S, and G2/M phases was then assessed via flow cytometry (FACSCalibur) using the FL3 channel (670 nm).

### 2.12. Wound-Healing Assay

Wound closure in CT-26 monolayers was quantified after 15 h treatment using Ibidi culture inserts (Culture-Insert 2 Well in μ-Dish 35 mm, Ibidi GmbH, Gräfelfing, Germany). Briefly, CT-26 cells were seeded at 3 × 10^4^ cells per well of the insert in 70 μL of complete medium and cultured overnight until confluent monolayers formed on both sides of the insert. The insert was then gently removed using sterile forceps, creating a defined 500 μm gap. Serum-free medium containing various formulations (CB: 200 nM; miR: 100 nM; R: 1.5 μM) or vehicle control was added. Cell migration was visualized before and after treatment using light microscopy (Olympus IX70, Tokyo, Japan), and the migration area was quantified using ImageJ software, v. 1.53t.

Relative migration area (% of area at 0 h) = 100% − [blank area_(15h)_/blank area_(0h)_ × 100%] (3)

### 2.13. Western Blotting

After 24 h treatment with various formulations (CB: 200 nM; miR: 100 nM; R: 1.5 μM) or vehicle control, total cellular proteins were extracted using radioimmunoprecipitation assay (RIPA) buffer. Protein concentrations were quantified using a BCA assay (Thermo Fisher, Waltham, MA, USA). Equal amounts of protein (30–50 μg per lane, normalized across samples) were separated by sodium dodecyl sulfate-polyacrylamide gel electrophoresis (SDS-PAGE), transferred to polyvinylidene fluoride (PVDF) membranes (Bio-Rad, Hercules, CA, USA), and probed with specific primary antibodies followed by horseradish peroxidase (HRP)-conjugated IgG secondary antibodies (JacksonImmunoResearch, West Grove, PA, USA). Protein bands were visualized using Immobilon enhanced chemiluminescence (Millipore, Billerica, MA, USA) and imaged with a Luminescence Imaging System (Amersham Imager 680; GE Healthcare, Chicago, IL, USA).

### 2.14. Macrophage–Cancer Cell Co-Culture and Cytokine Quantification

To evaluate the effect of formulations on TAM polarization, RAW 264.7 macrophages were co-cultured with CT-26 cells in cell culture inserts (Greiner Bio-One, Kremsmünster, Austria). Briefly, RAW 264.7 cells (1 × 10^5^ cells) were seeded in the upper insert chamber in 1.5 mL of DMEM complete medium, and CT-26 cells (2 × 10^5^ cells) were seeded in the lower well in 2.5 mL of RPMI-1640 complete medium. After 24 h of co-culture to establish cell–cell communication, CT-26 cells were treated with various formulations (CB: 200 nM; miR: 100 nM; R: 1.5 μM) for an additional 48 h at 37 °C. RAW 264.7 cells, CT-26 cells, and conditioned medium were collected for Western blot analysis.

### 2.15. Quantification of Cytokines and HMGB1

Cytokine and HMGB1 concentrations were determined using ELISA kits (BioLegend, San Diego, CA, USA). Each well was coated with 200 μL of capture antibody and incubated overnight, followed by blocking with assay diluent buffer for 1 h. Subsequently, standards and conditioned media were introduced and incubated for 2 h, followed by sequential incubation with detection antibodies (HMGB1, IL-10, TNF-α, TGF-β, IFN-γ), avidin-HRP, and substrate solutions (tetramethylbenzidine (TMB), provided in kit) for 15–30 min at room temperature in the dark until color development. Absorbance at 450 nm was measured using a TECAN ELISA reader to quantify protein levels.

### 2.16. Analysis of CRT and HMGB1 Localization

Following 24 h of treatment, cells were stained with ER-Tracker Green to visualize the endoplasmic reticulum, then fixed and blocked prior to antibody labeling. Primary antibodies against CRT or HMGB1 were applied and incubated overnight at 4 °C. The next day, Alexa Fluor 680- and Cy5.5-conjugated secondary antibodies were introduced to detect the respective targets. The cellular distribution of CRT and HMGB1 was assessed using CLSM on an Olympus FV10i system.

### 2.17. Measurement of Intracellular and Extracellular ATP

ATP levels were assessed using an ATP detection assay kit (Cayman, Ann Arbor, MI, USA) based on the luciferin-luciferase bioluminescence reaction. Intracellular ATP was quantified from lysed cells, whereas extracellular ATP was measured from conditioned media. Luminescence signals were recorded using a TECAN ELISA reader to evaluate ATP concentrations. The results are presented as percentage changes relative to untreated controls.

### 2.18. Hemolysis Assay

To assess hemocompatibility, rat red blood cells (RBCs) were collected into heparinized tubes (10 U/mL), washed three times with sterile PBS (pH 7.4) after centrifugation at 1000× *g* for 5 min at 4 °C, and resuspended in PBS to a final concentration of 2% (*v*/*v*). RBC suspensions (200 μL) were incubated with 200 μL of the formulations or 0.5% Triton X-100 (positive control, 100% hemolysis) or the PBS control (negative control, 0% hemolysis) for 24 h. Hemolysis was assessed by incubating supernatants from lysed RBCs with Drabkin’s reagent (Millipore Sigma) at 25 °C for 15 min in equal volume proportions (1:1, *v*/*v*). The intensity of the chromogenic signal was determined by measuring the optical absorbance at 540 nm with a TECAN microplate spectrophotometer.

### 2.19. Establishment of the CT-26 Tumor-Bearing Murine Model and Assessment of Antitumor Efficacy

BALB/c mice weighing approximately 20 g and aged 4–6 weeks were sourced from the National Laboratory Animal Center (Taipei, Taiwan). All in vivo procedures strictly adhered to ethical guidelines approved by the Institutional Animal Care and Use Committee (IACUC) of National Yang Ming Chiao Tung University (approval number: 1100411, 19 April 2021). Mice were subcutaneously implanted in the right flank with CT-26 cells (3 × 10^6^ cells suspended in 100 μL of PBS). When tumors reached ~60 mm^3^, mice (*n* = 3 per group) received the designated treatments twice weekly for 14 days via tail vein injection. The treatment groups were: (1) saline control (CTR), (2) miR/SLN-CSW, (3) CB, (4) CB/SLN-CSW, (5) CB + miR/SLN-CSW, (6) CB + miR + R/SLN-CSW, and (7) CB + miR + R/PGA-SLN-CSW, administered at 10 mg/kg for CB, 1.25 mg/kg for miR-142, and 6 mg/kg for R. Tumor dimensions were measured using digital calipers every 3–4 days (length and width in mm), and tumor volume was calculated using the modified ellipsoid formula:

V = (L × W^2^)/2 (4)

where L is the longest diameter and W is the shortest perpendicular diameter.

### 2.20. Positron Emission Tomography/Magnetic Resonance Imaging (PET/MRI)-Based Tumor Imaging

Tumor localization was evaluated using PET/MRI. Mice were fasted for at least 6 h before intravenous administration of [^18^F]-2-deoxy-2-fluoro-D-glucose (^18^F-FDG; 0.282 mCi per mouse, equivalent to approximately 10.43 MBq, diluted in 100 μL of sterile saline) on day 15. PET scans were acquired at 45 min post-injection using a Bruker 7T PET/MRI system (Bruker, Billerica, MA, USA), followed by MRI scans for anatomical reference and attenuation correction. Analysis of PET and MRI datasets was performed using AMIDE, an open-source imaging software, v. 1.0.6 (SourceForge, Ames, IA, USA).

### 2.21. Immune Profiling

Tumors and spleens from treated CT-26 tumor-bearing mice were harvested on day 15 (24 h after the final treatment), immediately placed in cold RPMI-1640 medium on ice, and processed into single-cell suspensions for characterization of tumor-infiltrating and immune cell populations. Single-cell suspensions (1 × 10^6^ cells in 100 μL of flow cytometry buffer) were stained with fluorescein-conjugated antibodies for 20 min in the dark. Flow cytometric analysis of dual-labeled cells stained with FITC- and PE-conjugated immune antibodies was conducted using FlowJo v10 software (FlowJo LLC, Ashland, OR, USA).

### 2.22. Multiplex Quantification of Cytokine and Chemokine Profiles

Circulating serum cytokine and chemokine levels were measured using a Bio-Plex multiplex assay (Bio-Rad, Hercules, CA, USA). Serum samples (50 μL, diluted 1:4 in assay diluent) and standards (provided in kit, creating 7-point standard curves) were incubated with antibody-coupled magnetic beads in a 96-well plate for 30 min at room temperature with shaking (300 rpm), washed three times using a magnetic washer, incubated with detection antibodies for 30 min, washed again, and incubated with streptavidin-phycoerythrin (SA-PE) for 10 min. After final washing, the beads were resuspended in assay buffer and analyzed using the Bio-Plex 200 system, which identifies individual beads by their spectral signature and quantifies bound PE fluorescence as a proportion of the analyte concentration. Analyzed targets included IL-1β, IL-2, IL-4, IL-5, IL-8 (CXCL-1), IL-10, IL-12, IL-17, interferon-gamma (IFN-γ), tumor necrosis factor-alpha (TNF-α), transforming growth factor-beta (TGF-β), and G-CSF.

### 2.23. Histology, Terminal Deoxynucleotidyl Transferase dUTP Nick End Labeling (TUNEL), and Immunohistochemitry (IHC)

Major organs including tumors, liver, kidneys, heart, spleen, and intestines (jejunum) were fixed in 4% paraformaldehyde, paraffin-embedded, sectioned into 4–5 μm thick sections, and stained with hematoxylin and eosin (HE). Stained samples were imaged using an Olympus VS120 slide scanner. Tumor apoptosis was assessed using a TUNEL assay (In Situ Cell Death Detection Kit, Roche, Basel, Switzerland), with imaging conducted using an Olympus FV10i confocal microscope with 488 nm excitation for TUNEL (green) and 405 nm for DAPI (blue), using a 20× objective. Tumor and spleen sections were subjected to IHC staining using primary antibodies targeting CD4, CD8, FoxP3, and PD-L1, followed by incubation with HRP-conjugated secondary antibodies. Signal development was performed using 3,3′-diaminobenzidine (DAB) with hematoxylin counterstaining. Stained sections were scanned using an Olympus VS120 slide scanner at 20× magnification, and digital images were analyzed. Quantitative assessment of DAB staining intensity was performed using Image Pro Plus 4.0 software.

### 2.24. Tumor Re-Challenge

Following the 14-day treatment regimen, primary tumors were surgically excised. Seven days post-surgery, CT-26 cells (1 × 10^6^ cells in 100 μL of PBS) were re-implanted into the contralateral flank of the same mice under brief isoflurane anesthesia to assess long-term immunological memory and protective immunity against tumor rechallenge. Tumor growth was monitored by caliper measurements biweekly over an additional 2 weeks. At the end of the study, the mice were sacrificed and their spleens were harvested, minced, and washed with PBS. Single-cell suspensions were stained with fluorescein-conjugated antibodies targeting T memory cell markers and analyzed via flow cytometry using FlowJo v10 software.

### 2.25. Hematology and Serum Biochemistry

Whole-blood samples were obtained via retro-orbital bleeding 48 h following the final treatment. Blood cell counts, including RBCs, white blood cells (WBCs), and platelets (PLTs), were quantified using a hematology analyzer (Sysmex XT-1800iv; Sysmex, Kobe, Japan). Serum was separated by centrifugation (1500 rpm, 20 min) and analyzed for biomarkers of hepatic (glutamic-pyruvic transaminase, GPT)), cardiac (creatine kinase-MB, CK-MB), and renal (blood urea nitrogen, BUN) function using specific assay kits (Fujifilm, Tokyo, Japan) and a Fuji Dri-Chem 7000V clinical chemistry analyzer (Fujifilm Corp., Tokyo, Japan).

### 2.26. Biodistribution Analysis

To quantify the tissue distribution of CB at 24 h post-injection, mice (*n* = 3 per group) were euthanized by CO_2_ asphyxiation, and tissue samples (tumors, liver, spleen, kidneys, and heart) were harvested, rinsed briefly in cold PBS to remove blood, blotted dry on filter paper, weighed (50–200 mg), and immediately homogenized with ice-cold methanol (~1 mL per 100 mg tissue) and distilled water (at a ratio of methanol:water:tissue = 4:1:1 *v*/*v*/*w*) using a tissue homogenizer, and then incubated on ice for 15 min to allow for complete protein precipitation and CB extraction. Following centrifugation of tissue homogenates at 12,000× *g* for 15 min at 4 °C, the supernatant containing CB was harvested and appropriately diluted with methanol. CB levels were then quantified spectrophotometrically at 280 nm using the Ultrospec 8000 PC spectrophotometer (Biochrom, Holliston, MA, USA) at a wavelength of 280 nm after the appropriate dilution with methanol.

### 2.27. Statistical Analysis

Comparative statistical analyses were performed using Student’s *t*-test, with results expressed as means ± standard deviations (SDs). Differences were considered statistically significant at * *p* < 0.05, with increasing levels of significance denoted by ** *p* < 0.01 and *** *p* < 0.001.

## 3. Results

### 3.1. Formulation and Physicochemical Profiling of SLNs Encapsulating CB, miR, and R

**Figure 1:**
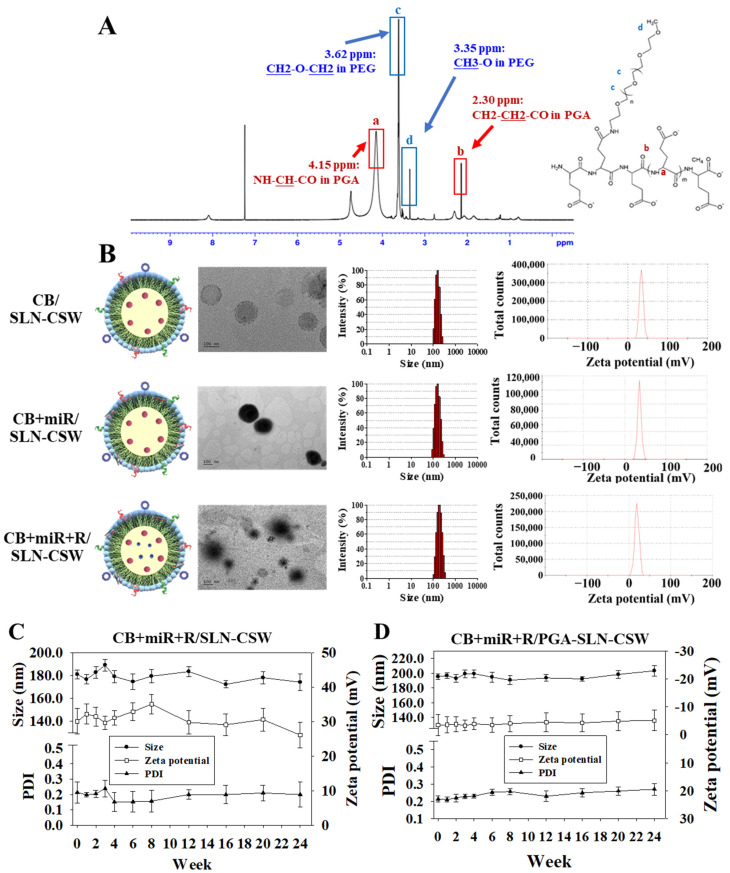
Physicochemical characterization and stability of SLN formulations. (**A**) Proton nuclear magnetic resonance (^1^H NMR) spectrum confirming PEG–PGA conjugation, with underlined peaks corresponding to specific proton environments: NH–CH–CO (a) and CH_2_–CH_2_–CO (b) in PGA; CH_2_–O–CH_2_ (c) and CH_3_–O (d) in PEG. (**B**) Transmission electron microscopy (TEM) images of CB/SLN-CSW, CB + miR/SLN-CSW, and CB + miR + R/SLN-CSW formulations, along with corresponding measurements of particle size, polydispersity index (PDI), and zeta potential (scale bar: 100 nm). (**C**) Stability profile of CB + miR + R/SLN-CSW over 24 weeks, showing changes in particle size, zeta potential, and PDI under storage conditions. (**D**) Stability profile of CB + miR + R/PGA-SLN-CSW over 24 weeks, showing changes in particle size, zeta potential, and PDI under storage conditions.

^1^H NMR spectroscopy confirmed the successful conjugation of PEG–PGA, with characteristic methylene proton peaks at 3.62 ppm and methyl proton peaks at 3.35 ppm, alongside methylene and methylidyne signals from PGA at 2.30 ppm and 4.15 ppm, respectively (Figure 1A). DSPE-PEG-peptides were synthesized via thiol–maleimide coupling of DSPE-PEG-maleimide with cysteine-containing C, S, or W peptides. Mass spectrometry verified molecular weights consistent with predicted values, confirming peptide conjugation (Figure S1A–C). Dynamic light scattering (DLS) revealed hydrodynamic diameters ≤ 200 nm for CB/SLN-CSW, CB + miR/SLN-CSW, and CB + miR + R/SLN-CSW (Figure 1B, Table 1), falling within the range considered optimal for passive and active accumulation in immune-excluded, MSS CRC tumors [7]. All formulations of SLN-CSW maintained narrow PDI values between 0.14 and 0.16, reflecting consistent homogeneity across batches. According to Table 1, the EE% remained high, ranging from 82.31–83.65% for CB, 89.54–89.56% for miR, and 85.18% for R. The corresponding DL% values were 19.79–20.99% for CB, 19.11–19.23% for miR, and 18.92% for R. Spherical morphology and uniform particle size distribution were confirmed by TEM, with no observable aggregation (Figure 1B). Each batch demonstrated high reproducibility (<5% variation) and excellent long-term stability at 4 °C over 24 weeks, showing negligible changes in size, zeta potential, or PDI (Figure 1C,D). These characteristics support the suitability and reproducibility of the formulations for preclinical MSS CRC models requiring extended formulation integrity.

### 3.2. pH-Responsive Features, Cellular Uptake, and Cytotoxicity

**Figure 2:**
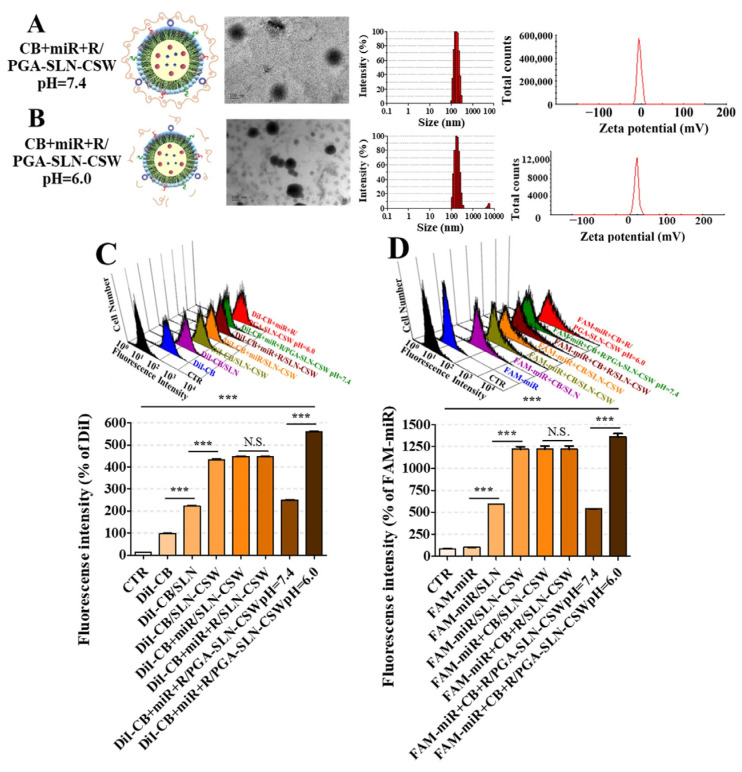

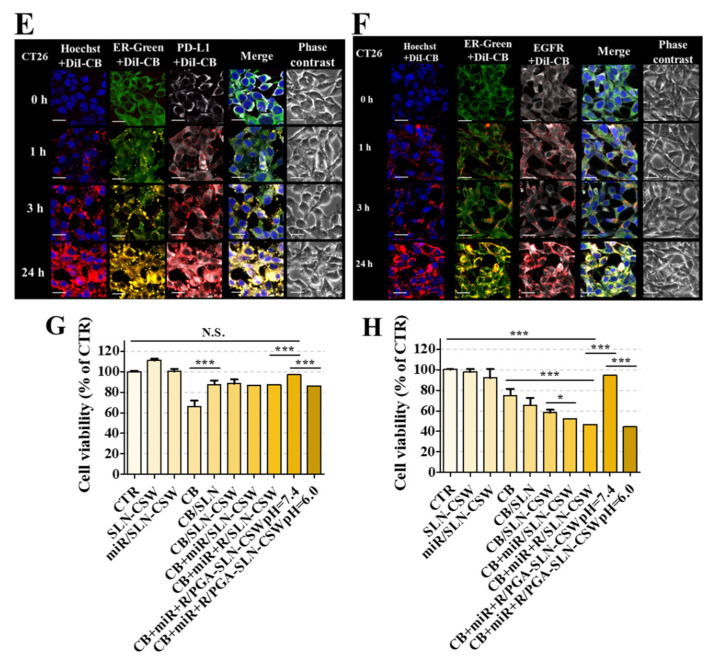
Assessment of cellular uptake, intracellular routing, and cytotoxic responses to CB-, miR-, and/or R-loaded SLN formulations. Particle morphology, size, and zeta potential of CB + miR + R/PGA-SLN-CSW were examined at pH 7.4 and 6.0 using TEM, revealing pH-responsive decoating of the PGA–PEG shell under acidic conditions ((**A**,**B**) scale bar: 100 nm). Intracellular fluorescence signals from DiI-labeled CB (0.5 μg/mL) and FAM-labeled miR-142 (100 nM) were quantified by flow cytometry in CT-26 cells under both pH conditions (**C**,**D**). The intracellular localization of DiI-CB + miR + R/SLN-CSW in CT-26 cells co-stained for PD-L1 or EGFR, along with ER and nuclear markers ((**E**,**F**) scale bar: 20 μm) was visualized using confocal laser scanning microscope (CLSM). Cytotoxicity was assessed in IEC-6 and CT-26 cells after 24 h of treatment using the sulforhodamine B assay (**G**,**H**). Statistical significance was indicated as * *p* < 0.05 and *** *p* < 0.001; N.S. = no significance.

The hydrodynamic diameter of CB + miR + R/PGA-SLN-CSW decreased from 195.26 ± 1.45 nm (pH 7.4) to 173.34 ± 3.89 nm (pH 6.0) and showed a zeta potential shift from −3.49 ± 0.23 mV to 19.78 ± 1.21 mV, consistent with PGA–PEG de-shielding in acidic environments (Figure 2A,B). This acid-triggered de-shielding leads to a more compact nanoparticle surface structure, which explains the reduction in hydrodynamic size and the marked increase in positive surface charge. TEM images showing uniform, non-aggregated spheres further support the stability and structural integrity of the nanoparticles under both pH conditions. Flow cytometry indicated enhanced DiI-CB uptake in CT-26 cells with SLN-CSW compared to unmodified SLNs or free drug, and further uptake enhancement at pH 6.0 for PGA-coated particles (Figure 2C). Similar trends were observed for FAM-miR delivery (Figure 2D). Uptake in IEC-6 cells at pH 7.4 was minimal (Figure S2A,B), suggesting tumor selectivity. Hemolysis assays confirmed non-lytic behavior for SLNs in contrast to free CB (11% hemolysis) (Figure S2C). Given that CT-26 cells recapitulate the limited immune infiltration characteristic of MSS CRC, with elevated PD-L1 and EGFR expression (Figure S3A), CLSM revealed that DiI-CB + miR/SLN-CSW rapidly associated with PD-L1 and EGFR within 1 h in CT-26 cells, mediated by W and C peptides. Subsequent trafficking to the ER was observed at 3 h via the S peptide, with intracellular retention sustained for up to 24 h (Figure 2E,F). Both PD-L1 and EGFR engagement were evident within 1 h, while FAM-miR + CB + R/SLN-CSW exhibited cytoplasmic localization by 8 h and remained detectable up to 24 h in CT-26 cells (Figure S3B,C). In IEC-6 cells, the PGA coating reduced CB-induced toxicity (Figure 2G). In CT-26 cells, CB/SLN-CSW increased cytotoxicity relative to free CB, and CB + miR-142 showed a combination index (CI) = 0.38. Treatment with triple-loaded SLN-CSW significantly reduced CT-26 cell viability to 46.68 ± 4.94%, with a further decline observed under acidic conditions (pH 6.0; Figure 2H and Figure S4A,B), aligning with the enhanced drug delivery capacity required to surmount the drug penetration barriers typically associated with MSS tumors.

### 3.3. ERS Induction via VCP Inhibition Promotes Both Autophagy and Apoptosis

**Scheme 2:**
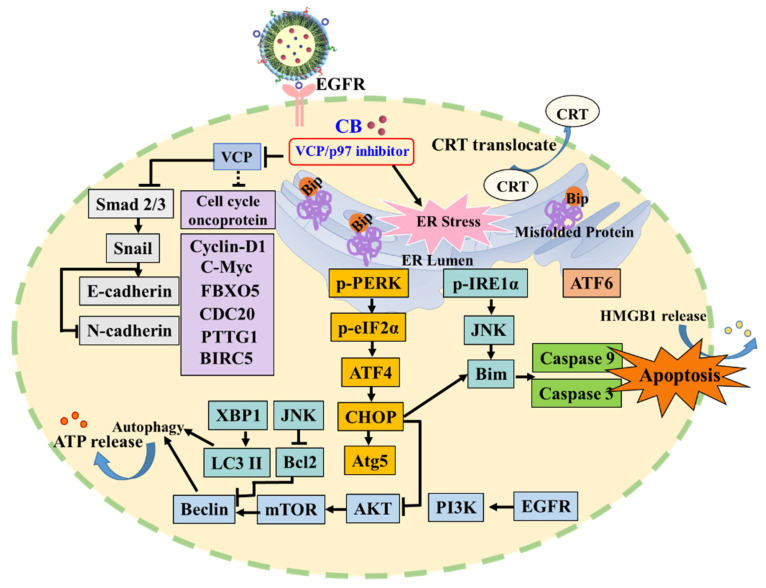
Schematic overview of ERS-related mechanisms in cancer illustrating how VCP/p97 inhibition modulates cell cycle regulators, epithelial–mesenchymal transition (EMT), autophagy, apoptosis, and ICD.

**Figure 3:**
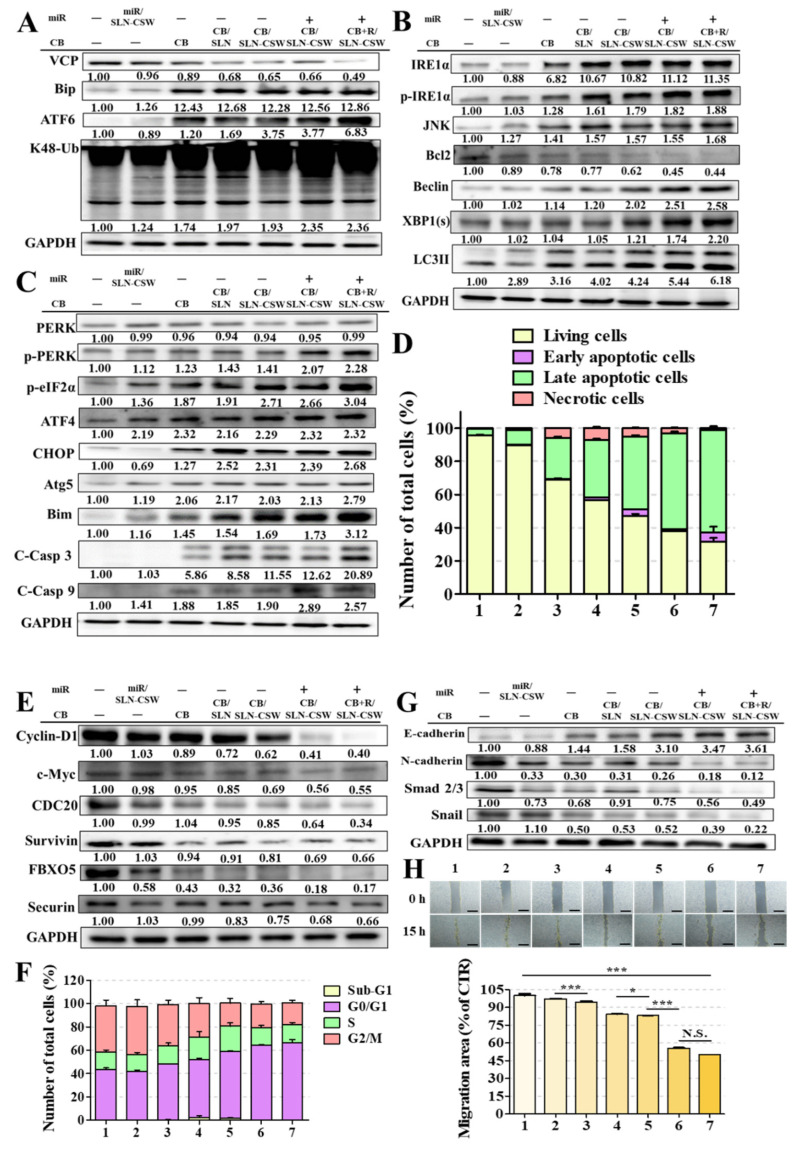
Induction of ERS-related pathways to trigger CT-26 cell death following 24 h treatment with various CB-, miR-, and/or R-loaded formulations in CT-26 cells. (**A**) Evaluation of the VCP-associated pathway. “−” indicates no addition of miR-SLN-CSW; “+” denotes the addition of miR-SLN-CSW. (**B**) Assessment of the IRE1α pathway. (**C**) Analysis of the PERK pathway. (**D**) Flow cytometric analysis of Annexin V and propidium iodide co-staining was used to quantify apoptotic and necrotic cell populations. (**E**) Examination of cell cycle oncoprotein expression. (**F**) Distribution of cell cycle phases. (**G**) Impact on the EMT pathway. (**H**) (**Up**) Migration assay results following 15 h treatment with various formulations. Scale bar, 250 µm. (**Down**) Quantification of the relative percentage of cell migration area post-treatment. Statistical significance was indicated as * *p* < 0.05 and *** *p* < 0.001; N.S. = no significance.

VCP is frequently overexpressed in CRC and associated with metastasis and progression [11], including in immune-cold MSS subtypes where proteostasis reliance is heightened. Its inhibition disrupts proteostasis by promoting misfolded protein accumulation in the ER, triggering ERS as indicated by Bip upregulation [12]. This activates the IRE1, ATF6, and PERK pathways, which regulate downstream proteins such as CHOP, Casp-3, Casp-9, and autophagy-related mediators, contributing to apoptotic cell death [7,13] (Scheme 2). Given the association of VCP overexpression with CRC progression [12], Western blot analyses showed that CB-containing formulations reduced VCP levels and elevated Bip, ATF6, and K48-linked ubiquitin, particularly in the CB + miR/SLN-CSW and CB + miR + R/SLN-CSW groups (Figure 3A). IRE1α signaling promoted autophagic responses (Atg5, Beclin, LC3II) through two branches: one involving XBP1s and LC3II, and the other mediated by JNK and Beclin (Figure 3B,C). Separately, the PERK pathway was distinctly activated, leading to phosphorylation of eIF2α (Figure 3C). This activation increased the translation of ATF4, which subsequently induced the expression of CHOP (Figure 3C). This pathway contributed to the upregulation of pro-apoptotic proteins including Bim, Casp-3, and Casp-9 (Figure 3C). These molecular events were also accompanied by a reduction in Bcl-2 expression (Figure 3B), reinforcing the dual induction of apoptosis and autophagy (Figure 3B,C). Annexin V/PI staining confirmed maximal apoptosis with CB + miR + R/SLN-CSW (Figure 3D). These findings indicated that targeted VCP inhibition not only disrupted proteostasis in MSS CRC cells but also initiated ERS-driven death pathways capable of reshaping the immune-resistant TME.

### 3.4. Suppression of EGFR Signaling, Cell Cycle Progression, and EMT in the MSS CRC Model

CT26 cells displayed elevated EGFR expression (Figure S3A) within an immune-inert microenvironment. A recent study also reported upregulation of EGFR and principal effectors of the PI3K/AKT/mTOR signaling cascade, consistent with hyperproliferative activity and enhanced survival signaling [1]. Treatment with CB-loaded SLNs (Figure S5) suppressed the oncogenic PI3K, p-AKT, and p-mTOR signaling cascade (Figure S5). The combination SLNs containing CB, miR, and R further downregulated key cell cycle regulators (Figure 3E). Notably, mitotic progression factors including securin, FBXO5, and CDC20 were significantly reduced, disrupting mitotic checkpoint control (Figure 3E). In parallel, the expression of proliferation drivers such as survivin, c-Myc, and cyclin D1 also decreased (Figure 3E), contributing to the induction of G0/G1 cell cycle arrest (Figure 3F). The most pronounced effects were observed in CB + miR + R/SLN-CSW-treated cells (Figure 3F). EMT reversal was evidenced by increased E-cadherin and reduced Smad2/3, Snail, and N-cadherin levels (Figure 3G), correlating with the impaired migration observed in the wound-healing assays (Figure 3H).

### 3.5. ERS-Driven ICD and Immune Modulation

**Figure 4:**
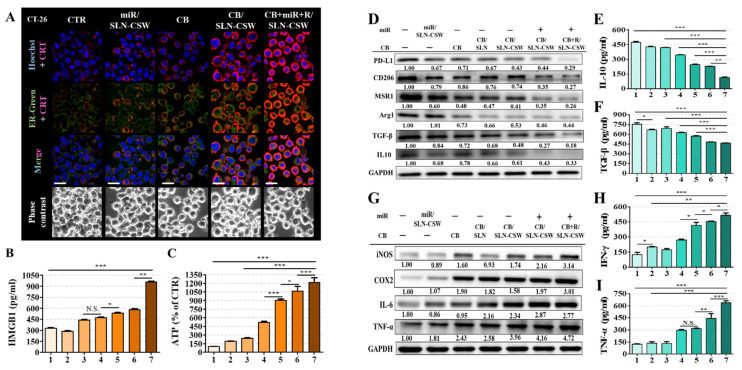
Evaluation of ICD hallmarks and immune responses after 24 h treatments with CB-, miR-, and/or R-loaded formulations. (**A**) CLSM imaging of effect of CRT exposure on cell membranes (blue: Hoechst; green: ER; gray: CRT). Scale bar: 20 µm. (**B**,**C**) ELISA-based quantification of HMGB1 secretion and ATP release. (**D**–**F**) Western blot and ELISA analyses of M2-associated markers (CD206, MSR1, Arg1) and immunosuppressive cytokines (IL-10, TGF-β). (**G**–**I**) Assessment of M1-related proteins (iNOS, COX-2) and proinflammatory cytokines (IFN-γ, TNF-α) via Western blot and ELISA (N.S., no significance; statistical significance at * *p* < 0.05, ** *p* < 0.01, and *** *p* < 0.001).

This study provided mechanistic insight into CB as a type II ICD inducer. In CT-26 cells, which recapitulate these MSS-associated immune barriers, CB directly activated ERS to initiate ICD, as confirmed by Western blot analysis (Figure 3A–C). Consistent with the stress-prone phenotype of MSS CRC, VCP ablation triggered ROS production, validated by DCFH-DA staining quantification via flow cytometry and CLSM (Figure S6A,B). This led to the accumulation and release of DAMPs—CRT, HMGB1, and ATP—from tumor cells (Figure 4A–C and Figure S7), events that can help breach the immune-exclusive TME. These alterations coincided with a decrease in the secretion and/or expression of immunosuppressive molecules including MSR1, CD206, IL-10, TGF-β, Arg1, and PD-L1 (Figure 4D–F), and increased proinflammatory mediators (TNF-α, IFN-γ, IL-6, iNOS, COX-2) (Figure 4G–I), indicating restoration of an inflamed phenotype. Notably, CB + miR + R/SLN-CSW markedly suppressed IL-10 and TGF-β (Figure 4E,F) while increasing IFN-γ and TNF-α (Figure 4H,I), changes particularly relevant to reversing the MSS CRC immunosuppressive milieu.

### 3.6. CB/miR/R-Incorporated Constructs Reshaped the MSS CRC TME by Enhancing Antigen-Presenting Cell Activation, Promoting Cytotoxic Lymphocyte Recruitment, and Downregulating Immunosuppressive Treg and TAM Populations

**Figure 5:**
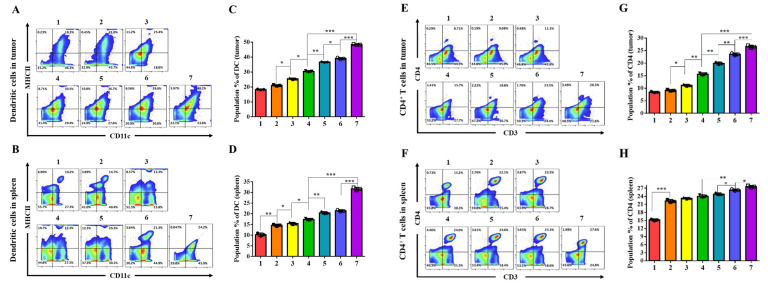

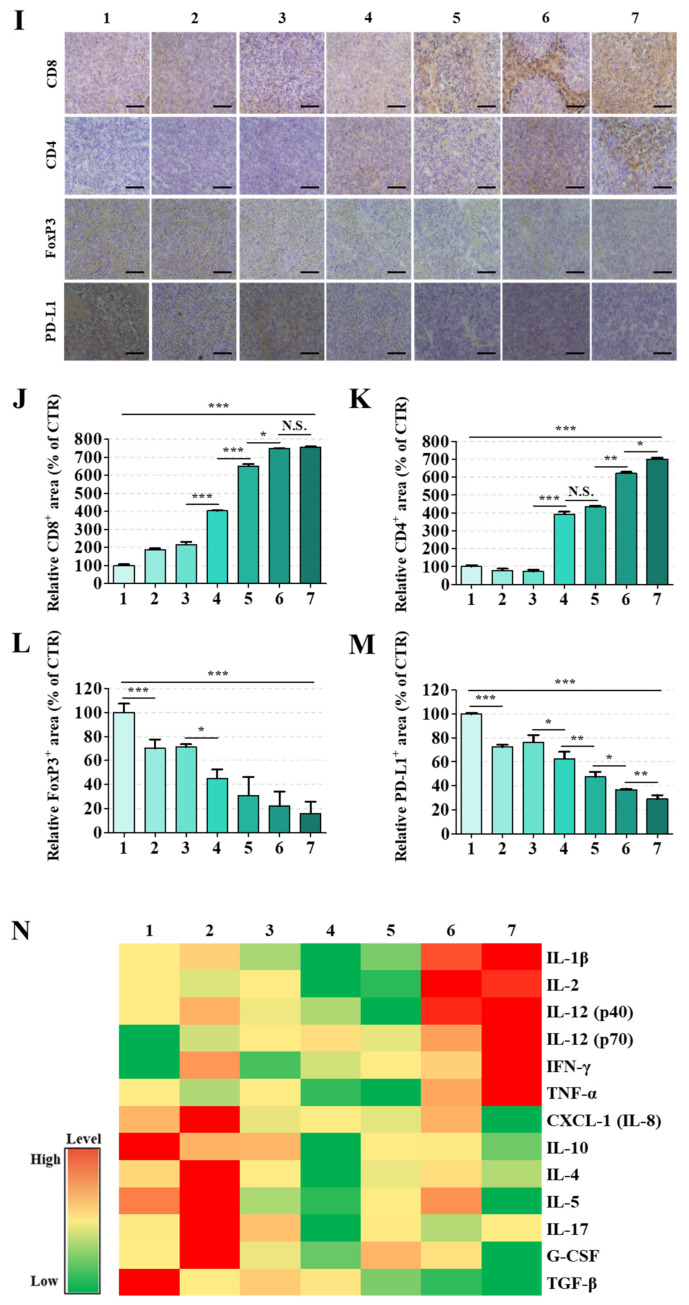
Evaluation of antitumor immune responses after 14 days of in vivo administration of CB (10 mg/kg), miR (1.25 mg/kg), and/or R (6 mg/kg) via tail vein injections twice per week. Panels (**A**–**D**) show flow cytometric results and quantification of MHC II-positive dendritic cells in the tumor (**A**,**C**) and spleen (**B**,**D**). Panels (**E**–**H**) present flow cytometric data and counts of CD4+ T cells within the tumor (**E**,**G**) and spleen (**F**,**H**). Panel (**I**) provides immunohistochemical images displaying the spatial distribution of CD8+, CD4+, regulatory T cells, and PD-L1 expression in tumor tissue sections, with a scale bar of 200 µm. Quantitative analyses of the IHC-stained regions for CD8+ T cells (**J**), CD4+ T cells (**K**), Tregs (**L**), and PD-L1 (**M**) are depicted. Statistical significance in all bar graphs was indicated as * *p* < 0.05, ** *p* < 0.01, and *** *p* < 0.001; N.S. = no significance. Panel (**N**) shows the serum cytokine and chemokine levels determined by the Bio-Plex Pro Mouse Cytokine Plex Immunoassay. Treatment groups are labeled as follows: 1: CTR; 2: miR/SLN-CSW; 3: CB; 4: CB/SLN-CSW; 5: CB + miR/SLN-CSW; 6: CB + miR + R/SLN-CSW; 7: CB + miR + R/PGA-SLN-CSW (*n* = 3).

Considering the frequent dysfunction of dendritic cell activation and limited infiltration of effector T cells in MSS CRC, the therapeutic impact of formulations containing CB alone or in combination with immunomodulatory miR-142 and R was examined in CT-26 tumor-bearing mice (Figure 5 and Figure S8–S11). All SLN-CSW-based treatments, especially CB + miR + R, promoted DC maturation and expansion of CD11c^+^MHCII^+^ populations, with the PGA-coated formulation yielding the most substantial effect (Figure 5A–D), consistent with ICD-driven antigen presentation (Figure 4A–C). CB + miR + R/SLN-CSW, with or without PGA, markedly increased intratumoral infiltration of CD4^+^ helper and CD8^+^ cytotoxic T cells (Figure 5E–H and Figure S8), a hallmark typically diminished in MSS CRC, while concomitantly reducing regulatory T cells expressing CD25 and FoxP3, and TAMs marked by F4/80 and CD206 (Figures S9–S11). The concurrent increase in CD8^+^ and CD4^+^ T cell populations and reduction in PD-L1 and TAM markers in tumor and spleen tissues (Figure 5I–M and Figure S11A–D) indicates reversal of the TAM-enriched TME. Cytokine analysis demonstrated a transition favoring a proinflammatory milieu, characterized by increased levels of IL-1β, IL-2, IL-12, IFN-γ, and TNF-α, alongside a reduction in immunosuppressive mediators such as TGF-β, IL-4, IL-5, IL-10, and CXCL-1, particularly in the group receiving the triple combination within PGA-SLN-CSW (Figure 5N).

### 3.7. Antitumor Efficacy of pH-Responsive CB + miR + R/PGA-SLN-CSW Nanoformulation in MSS CRC Validated Through Integrated Imaging Modalities and Tissue-Level Analysis

**Figure 6:**
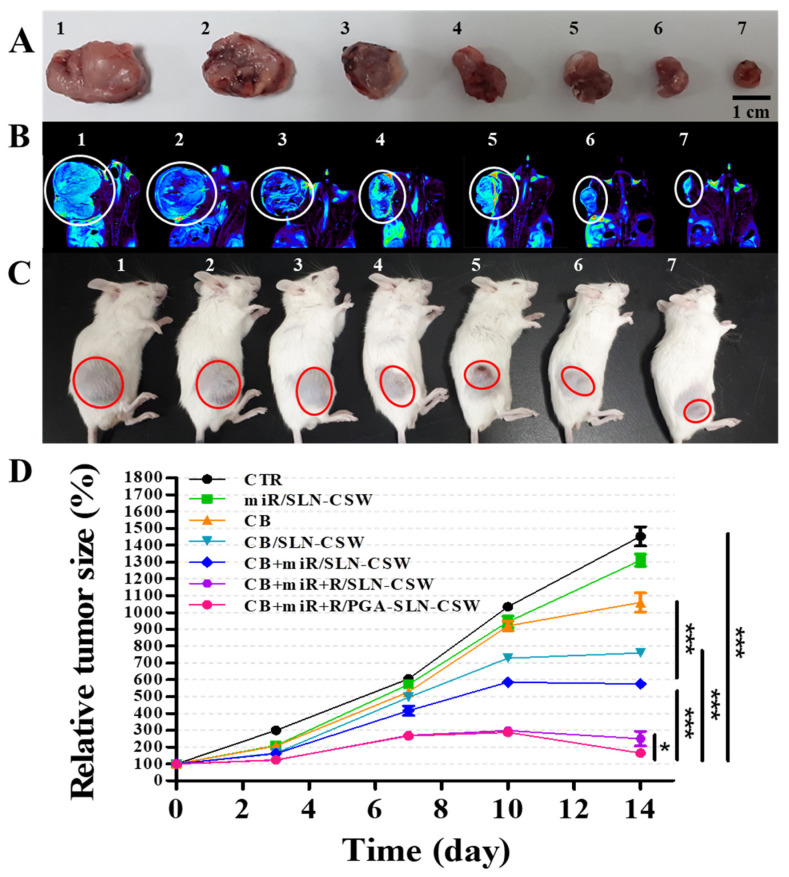

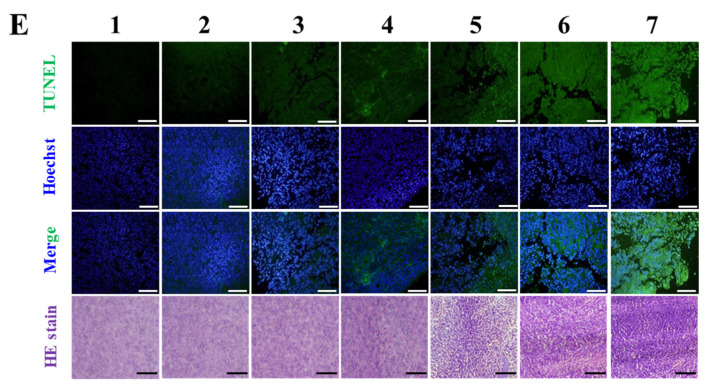
Therapeutic outcomes of CB/miR/R formulations in CT26-bearing mice with MSS CRC. (**A**) Tumors isolated from CT-26 tumor-bearing mice following a 14-day treatment regimen. (**B**) PET/MR images of CT-26-bearing mice obtained after 14 days of therapy using the radiant probe [^18^F]-2-deoxy-2-fluoro-D-glucose (^18^F-FDG, 0.282 mCi), with tumor regions highlighted by white circles. (**C**) Images of CT-26-bearing mice after 14-day treatment, with visible tumor sites indicated by red circles. (**D**) Tumor volume measurements were conducted with digital calipers every 4 days during the 14-day therapy (statistical significance indicated as * *p* < 0.05 and *** *p* < 0.001. (**E**) TUNEL analysis (upper panel) after a complete 14-day therapy, demonstrating in vivo apoptosis in tumor cells (green), with nuclei (blue) stained using Hoechst. Scale Bar: 200 µm. Representative micrographs of tumor tissue sections stained with hematoxylin and eosin (HE) demonstrating histological architecture. Scale Bar: 200 µm. 1: CTR; 2: miR/SLN-CSW; 3: CB; 4: CB/SLN-CSW; 5: CB + miR/SLN-CSW; 6: CB + miR + R/SLN-CSW; 7: CB + miR + R/PGA-SLN-CSW (*n* = 3). Representative images from five independent samples are presented.

In light of the intrinsic immunoresistance of MSS CRC, achieving tumor regression in CT26-bearing mice sets a demanding benchmark for therapeutic performance. Treatment with the CB, miR, and R formulation delivered via the PGA-SLN-CSW platform significantly reduced tumor volume compared with all other groups (Figure 6A–D), which was attributed to selective activation of the PGA coating in the acidic TME, which enhances drug release and promotes immunomodulatory effects within MSS CRC lesions. PET/MRI demonstrated reduced ^18^F-FDG uptake (Figure 6B), and optical imaging confirmed tumor shrinkage (Figure 6C). Histopathology showed extensive TUNEL positivity and pyknosis (Figure 6E), features indicative of apoptosis induction in immune-refractory MSS tumors.

### 3.8. The Multifunctional Formulation-Induced Immunological Recall and Shift of TAMs Toward Antitumor Function in MSS CRC

**Figure 7:**
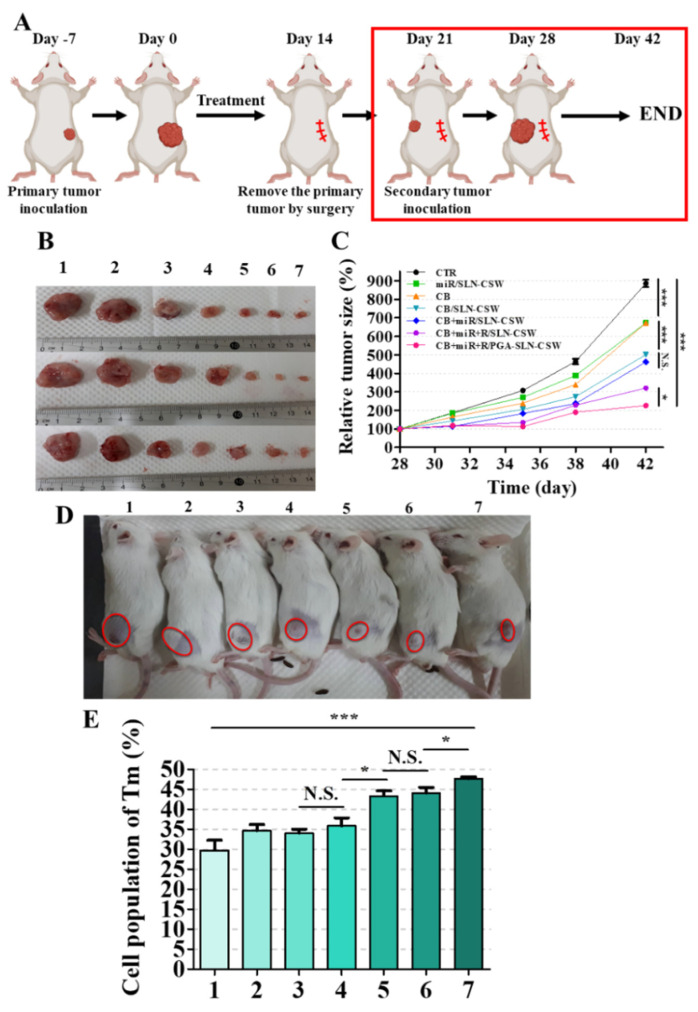
Protective immunity and memory T cell responses in tumor rechallenge model. (**A**) Experimental schematic depicting rechallenge study design. The red square indicates the scheduled period for secondary tumor treatment. (**B**) Macroscopic evaluation of secondary tumors excised from rechallenged mice. (**C**) Temporal analysis of secondary tumor growth measured by digital calipers at biweekly intervals over a 14-day period. (**D**) Representative images of rechallenged tumor-bearing mice at study termination, with visible tumor sites indicated by red circles. (**E**) Comparative analysis of relative effector memory T cell populations across treatment groups. Statistical significance indicated as * *p* < 0.05 and *** *p* < 0.001; N.S. = no significance. 1: CTR; 2: miR/SLN-CSW; 3: CB; 4: CB/SLN-CSW; 5: CB + miR/SLN-CSW; 6: CB + miR + R/SLN-CSW; 7: CB + miR + R/PGA-SLN-CSW (*n* = 3).

Post-surgical re-challenge in CT-26-bearing mice (Figure 7A) revealed that CB + miR + R/PGA-SLN-CSW limited secondary tumor outgrowth, maintaining the smallest volumes compared to controls (Figure 7B–D). The T cell subset with effector memory function in the spleen (CD62L^−^CD44^+^) progressively expanded following treatment with CB, miR, and R, reaching their highest levels within the PGA-coated formulation group (Figure 7E). In MSS CRC, where immune memory formation is typically compromised by a suppressive TME, these findings highlight the platform’s potential to induce durable antitumor immunity capable of preventing recurrence.

### 3.9. Biosafety and Biodistribution Profiles Affirmed Favorable Systemic Tolerance in the MSS CRC Model

**Figure 8:**
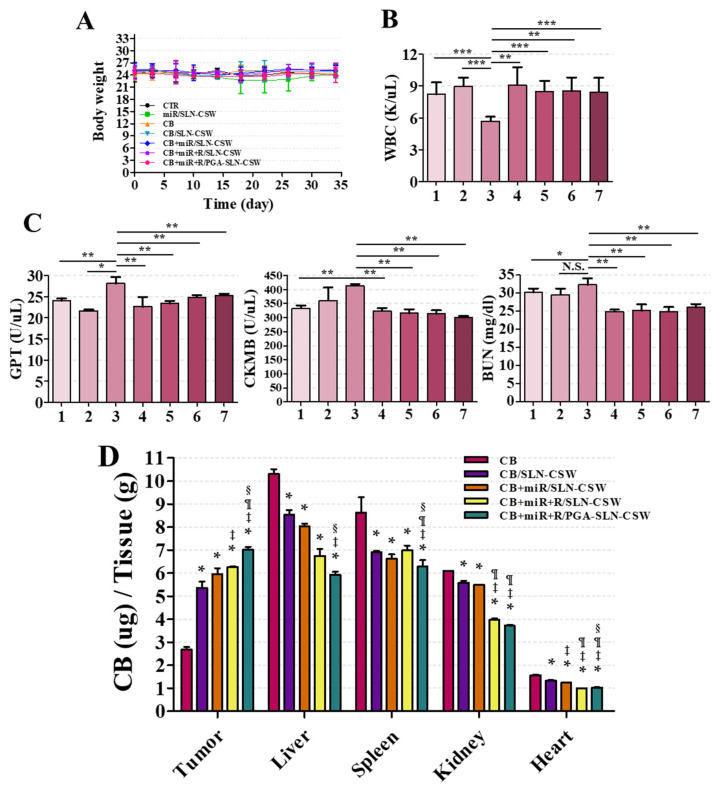
Biosafety and biodistribution studies of a series of formulations integrating CB, miR, and/or R in CT26-bearing mice. (**A**) Body weight was monitored bi-weekly over 14 days. (**B**) WBC counts following 14-day treatment. (**C**) Serum levels of GPT, BUN, and CK-MB were measured 48 h after final administration (N.S., no significance; statistical significance: * *p* < 0.05, ** *p* < 0.01, *** *p* < 0.001). (**D**) CB biodistribution across organs after 14-day treatment, quantified via UV spectrophotometry in CT26-bearing mice. Statistical comparisons using Student’s *t*-test analysis: * *p* < 0.05 compared with CB; ^‡^ *p* < 0.05 compared with CB/SLN-CSW; ^¶^ *p* < 0.05 compared with CB + miR/SLN-CSW, and ^§^ *p* < 0.05 compared with CB + miR + R/SLN-CSW. Treatment groups: 1: CTR; 2: miR/SLN-CSW; 3: CB; 4: CB/SLN-CSW; 5: CB + miR/SLN-CSW; 6: CB + miR + R/SLN-CSW; 7: CB + miR + R/PGA-SLN-CSW.

The SLN-based formulations exhibited favorable systemic safety. Over the 42-day period, they consistently preserved body weight (Figure 8A), maintained hematological indices near baseline levels (Figure 8B and Figure S12), and mitigated biochemical signs of organ toxicity evident in the free CB-treated group (Figure 8C). Histopathological analysis demonstrated reduced off-target tissue damage compared to free drug administration (Figure S13). Biodistribution studies revealed maximal tumor-specific accumulation alongside minimal hepatic and splenic deposition for PGA-coated SLNs co-loaded with CB, miR, and R (CSW) (Figure 8D), suggesting effective targeted delivery to the immune-cold MSS CRC microenvironment.

## 4. Discussion

The CT26 model holds translational importance in CRC research as it recapitulates the MSS, mismatch repair–proficient phenotype that dominates the clinical landscape [1,2]. These tumors exhibit poor cytotoxic T lymphocyte infiltration, enrichment of immunosuppressive TAMs, and a cytokine milieu typified by IL-17, G-CSF, and CXCL-1, which collectively reinforce immune exclusion and therapeutic refractoriness [1,3]. The resulting TME, compounded by a dense extracellular matrix and limited drug permeability, presents formidable biological and physical barriers to therapeutic efficacy. These immunosuppressive and structural features underlie the poor responsiveness of MSS CRC to immune checkpoint inhibitors (ICIs) and highlight the need for therapeutic systems capable of remodeling the TME while preserving immune competence.

To address these challenges, a pH-sensitive PEG–PGA-coated, PD-L1/EGFR-targeted SLN system was engineered to co-deliver the proteostasis modulator CB, the immune-regulatory miR-142, and the TLR7 agonist R. The optimized formulations exhibited hydrodynamic diameters below 200 nm, a narrow PDI (~0.15), and encapsulation efficiencies above 80% (Table 1), parameters favorable for passive and receptor-mediated tumor accumulation. TEM imaging confirmed a uniform spherical morphology without aggregation, while reproducibility analyses across independent batches demonstrated a <5% variation in size, ζ-potential, and drug loading, underscoring both formulation consistency and scalability. Long-term storage at 4 °C for 24 weeks showed no detectable change in particle size, surface charge, or polydispersity (Figure 1C,D), further supporting suitability for extended preclinical use. Collectively, these results validate the structural integrity, batch-to-batch consistency, and translational readiness of this multifunctional nanoplatform.

Building on these physicochemical attributes, the acid-labile PEG–PGA coating was designed to conceal targeting ligands at physiological pH while enabling de-shielding and payload release within the acidic TME (Figure 2). PD-L1- and EGFR-binding peptides facilitated dual targeting of tumor cells and TAMs, whereas the ER-homing S peptide directed intracellular trafficking toward stress-regulatory compartments [7,14,15]. This hierarchical targeting strategy ensured that proteostasis disruption, checkpoint modulation, and innate immune activation occurred selectively within tumor tissues, thereby overcoming both immune resistance and physical exclusion barriers inherent to MSS CRC. Beyond confirming formulation precision and translational feasibility, these data establish a rational foundation for clinically adaptable, ER-targeted nanotherapeutics aimed at reinstating immune vigilance in CRC and other immune-refractory malignancies.

CB-5083, a first-in-class ATP-competitive VCP/p97 inhibitor, demonstrated potent preclinical anticancer activity but showed reversible visual toxicity resulting from off-target phosphodiesterase-6 (PDE6) inhibition [16]. Other VCP inhibitors and proteostasis modulators, including ML240 and allosteric agents such as NMS-873, represent promising alternatives and further validate VCP inhibition as a viable therapeutic strategy when off-target liabilities are managed [17,18]. Frequent VCP overexpression in CRC [11] suggests that dysregulated proteostasis contributes to malignant persistence and stress tolerance. CB, initially identified as a VCP inhibitor, elicited Bip-dependent ER stress in CRC cells, sequentially engaging IRE1, ATF6, and PERK branches that culminate in CHOP activation, Casp-3/9 cleavage, and LC3II/Beclin accumulation—hallmarks of apoptosis and autophagy [7,12,13]. Extending this framework, the nanoformulations incorporating CB, miR, and R further intensified ER dysfunction and disrupted adaptive stress recovery in CT-26 cells (Figure 3). VCP inhibition interfered with Bip activity and K48-linked ubiquitination, diminishing protein-folding capacity while constraining ATF6-driven transcriptional compensation. Concurrent modulation of the IRE1α–XBP1s and PERK–eIF2α/ATF4–CHOP cascades reflected sustained translational arrest and apoptotic signaling, whereas the JNK–Beclin axis mediated LC3II accumulation, which are consistent with autophagic engagement under prolonged stress.

Beyond proteostatic failure, these events intertwined with transcriptional and signaling alterations that compromise tumor cell fitness. Downregulation of cyclin D1, c-Myc, CDC20, Survivin, FBXO5, and Securin (Figure 3E) suggested a collapse of proliferative control networks known to uphold genomic stability and chemoresistance [19,20,21]. The PGA–SLN–CSW system co-delivering CB, miR, and R further reinforced this blockade, as reflected by the observed G0/G1 arrest (Figure 3F). Attenuation of EGFR, PI3K, p-AKT, and p-mTOR (Figure S5) implied additional disruption of oncogenic signaling that ordinarily restrains ER stress and sustains immune evasion [22,23]. VCP inhibition also curtailed EMT progression, lowering Smad2/3, Snail, and N-cadherin while restoring E-cadherin (Figure 3G), thereby limiting the migratory potential (Figure 3H). Collectively, these mechanistic layers depict a network in which impaired protein quality control, restrained growth signaling, and ER stress converge to activate apoptotic and autophagic death programs mediated by CHOP, Casp-3/9, and LC3II/Beclin [12,13,24].

The triple-payload nanoformulation co-delivering CB, miR, and R elicited the most pronounced cell cycle arrest, with maximal G0/G1 accumulation observed in CB + miR + R/SLN-CSW-treated cells (Figure 3F). These responses led to ICD, with CRT exposure, ATP release, and HMGB1 liberation (Figure 4A–C), thereby promoting antigen presentation and effector T cell priming [24,25]. The delivery of miR-142 downregulated PD-L1, promoted M1-like macrophage polarization (Figure 4D), suppressed EMT by reducing Smad2/3 and Snail expression, and restored E-cadherin, limiting tumor migration (Figure 3G,H). R-mediated TLR7 activation induced DC maturation and pro-inflammatory cytokine production (Figure 4D–I), further amplifying innate and adaptive antitumor responses (Figure 5). These findings suggest that coordinated targeting of proteostasis, checkpoint axis regulation, and TLR7 signaling not only dismantles EGFR-driven oncogenic survival circuitry but also reshapes the TME into a niche favoring T cell recruitment and immune activation, providing a mechanistically substantiated strategy to surmount immunotherapy resistance in MSS CRC.

The MSS CRC TME was transformed from an immune-restrictive to a T cell-permissive state through these convergent mechanisms in vivo. Treatment with CB + miR + R/PGA-SLN-CSW increased cytotoxic (CD8^+^) and helper (CD4^+^) T cell populations, reduced FoxP3^+^ regulatory T cells, and depleted CD206^+^ M2-type TAMs within the tumor and/or spleen (Figure 5A–M and Figure S8–S11). Serum cytokine profiling revealed elevated TNF-α, IFN-γ, IL-2, and IL-12, concurrent with reductions in IL-17, TGF-β, and G-CSF (Figure 5N), indicating attenuation of TAM recruitment and immunosuppression. This dual modulation of immune activation and TAM reprogramming targeted key resistance pathways in MSS CRC. In the MSS CRC setting, combination regimens incorporating CB, miR-142, and R effectively suppressed tumor burden and established long-lasting immune memory, as indicated by a durable response to tumor rechallenge and persistent effector memory T cell populations (Figure 7). These findings suggest that combination therapy with this nanoplatform could elicit enduring antitumor immunity in tumors otherwise refractory to current immunotherapies. The tumor-selective delivery profile minimized systemic toxicity, addressing significant limitations of free CB and R [8,9,26].

Treated mice maintained stable body weights, normal hematologic and biochemical indices, and intact organ histology, while biodistribution studies confirmed preferential tumor accumulation with reduced reticuloendothelial uptake (Figure 8, Figures S12 and S13). Although tumor-targeting peptides like our PD-L1-, EGFR-, and ER-targeting motifs are typically less immunogenic than protein biologics [5,18], antibody or T-cell responses can still occur, especially with repeated dosing or modified sequences. Recent studies have demonstrated that rational design can minimize this risk, but careful immunogenicity evaluation remains critical prior to clinical translation [27]. Furthermore, serum biochemistry analysis (Figure 8C) provides critical evidence for organ-selective delivery. While free CB induced significant hepatic (GPT: *p* < 0.05) and cardiac (CK-MB, *p* < 0.01) stress, nanoformulated CB eliminated these toxicity signals while achieving superior tumor control, supported by improved tumor accumulation over organs (biodistribution data; Figure 8D). This demonstrates that targeted delivery can rescue VCP inhibitors—abandoned after CB clinical failure due to off-target toxicity [16]—by fundamentally altering the biodistribution to achieve tumor selectivity. These findings establish a favorable therapeutic window that meets ICH S9 regulatory requirements and provide a biomarker framework for clinical monitoring in future Phase I trials.

## 5. Conclusions

**Scheme 3:**
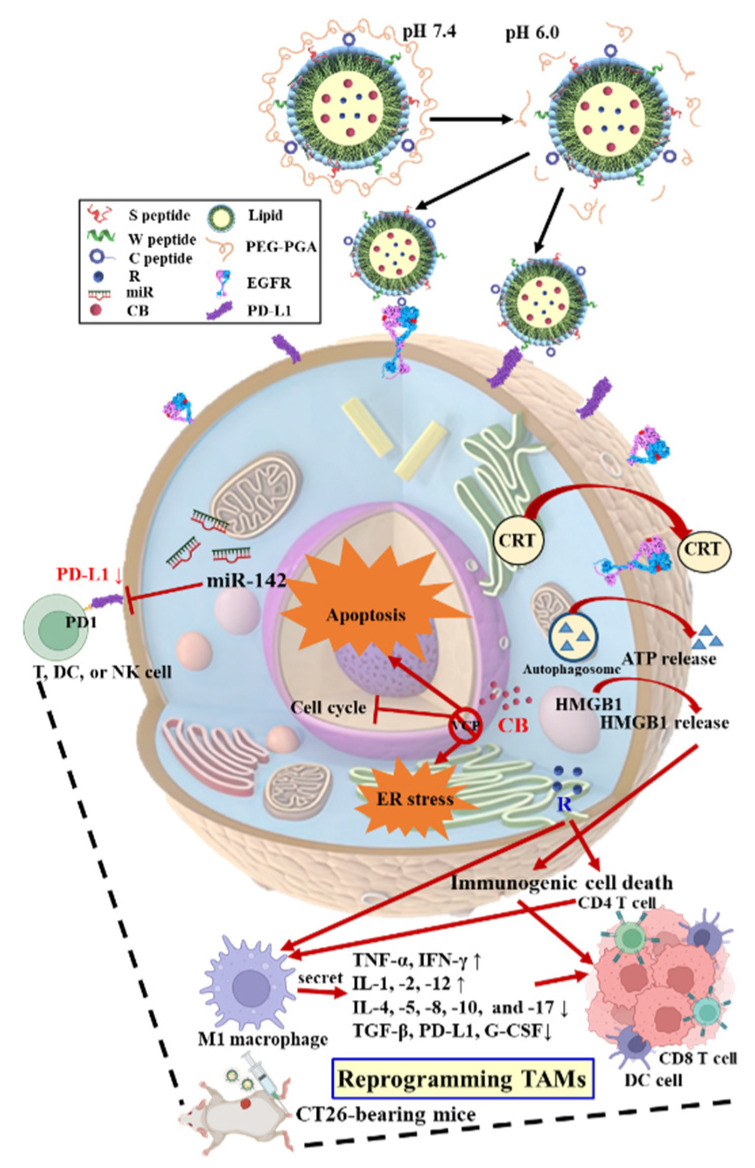
Schematic representation of the PEG–PGA-coated, PD-L1/EGFR-targeted SLN platform for immune reprogramming in MSS CRC. The pH-sensitive PEG–PGA shell masks target ligands in circulation and detach in the acidic TME, enabling PD-L1/EGFR peptide-mediated tumor and TAM targeting. An ER-homing S peptide directs co-delivered CB, miR-142, and R to stress-regulatory compartments. CB induces sustained ER stress via IRE1α/XBP1s, JNK/Beclin, and PERK/ATF4/CHOP signaling, triggering apoptosis, autophagy, and ICD (CRT exposure, ATP release, HMGB1 liberation). miR-142 downregulates PD-L1, promotes M1-like macrophage polarization, and inhibits EMT via Smad2/3–Snail suppression. R activates TLR7, driving DC maturation and pro-inflammatory cytokine production. In vivo, these mechanisms convert the immune-excluded TME into a T cell-permissive state, increasing CD8^+^ and CD4^+^ T cell infiltration, reducing FoxP3^+^ Tregs, and depleting CD206^+^ M2 TAMs. Preferential tumor accumulation with minimal systemic toxicity confers durable immune memory and tumor rechallenge resistance. The modular design enables adaptation to other TAM-rich, immune-excluded malignancies such as pancreatic ductal adenocarcinoma.

This study establishes a pH-responsive, PD-L1/EGFR-targeted, ER-homing SLN platform co-delivering CB, miR, and R as a potent strategy to reprogram the immunosuppressive microenvironment of MSS CRC. The optimized nanoformulation demonstrated favorable physicochemical characteristics and long-term stability. Mechanistically, CB + miR + R/PGA-SLN-CSW treatment activated all major ER stress pathways (BiP, PERK, IRE1α, ATF6, CHOP), leading to apoptosis and immunogenic cell death, as evidenced by CRT exposure, HMGB1 release, and ATP secretion. These effects collectively reshaped the tumor immune landscape, promoting CD8^+^ T cell infiltration, increasing the CD8^+^/FoxP3^+^ ratio, enhancing M1-like polarization of TAMs, and elevating systemic pro-inflammatory cytokines. Therapeutically, the treatment achieved marked tumor growth suppression and induced durable immune protection, as shown by resistance to tumor rechallenge and increased memory T cell populations. Safety assessments revealed no systemic toxicity, with normal organ function markers (GPT, CK-MB, BUN), hematological parameters, and histopathology. Biodistribution analysis confirmed preferential tumor accumulation, supporting the observed efficacy and safety profile. While further validation in additional models and extended treatment timelines is warranted, these findings demonstrate that integrating proteostasis disruption, immune checkpoint modulation, and innate immune activation within a multi-targeted nanodelivery system can effectively convert immunologically inert MSS CRC into a responsive state. This platform offers a promising foundation for clinical translation and may be broadly applicable to other TAM-enriched, immunotherapy-resistant malignancies (Scheme 3).

## Figures and Tables

**Table 1:** Physicochemical characteristics, encapsulation efficiency, and batch reproducibility of multifunctional SLNs.

Formulations	Size (nm)	PDI ^a^	Zeta Potential (mV)	EE% ^b^ of CB	EE% of miR	EE% of R	DL% ^c^ of CB	DL% of miR	DL% of R ^d^	Batch-to-Batch Variation (%) ^e^	Stability (ΔSize/ΔPDI @ 24 wk) ^f^
**CB/SLN-CSW**	174.1 ± 1.71	0.15 ± 0.01	36.03 ± 0.67	82.31 ± 0.98	--	--	19.79 ± 0.67	--	--	<5	<3/<2
**CB+miR/SLN-CSW**	177.57 ± 1.86	0.14 ± 0.01	31.7 ± 0.72	83.47 ± 1.98	89.56 ± 0.23	--	20.78 ± 0.54	19.11 ± 1.21	--	<5	<3/<2
**CB+miR+R/SLN-CSW**	182.47 ± 0.81	0.16 ± 0.01	29.83 ± 0.65	83.65 ± 0.87	89.54 ± 0.12	85.18 ± 0.31	20.99 ± 0.43	19.23 ± 0.04	18.92 ± 0.67	<5	<3/<2

^a^ Polydispersity indexes. ^b^ Encapsulation efficiency. ^c^ Drug-loading capacity. ^d^ All data represent mean ± SD (n = 3 batches). ^e^ Batch-to-batch variation <5% for all parameters. ^f^ Stability variation data from Figure 1C,D.

## Data Availability

The original contributions presented in this study are included in the article/Supplementary Material. Further inquiries can be directed to the corresponding author.

## Supplementary Materials

The following supporting information can be downloaded at: https://www.mdpi.com/article/10.3390/pharmaceutics17111503/s1, Figure S1: Mass spectra of (A) DSPE-PEG-C, (B) DSPE-PEG-S, and (C) DSPE-PEG-W; Figure S2: Quantification of intracellular fluorescence intensity of (A) DiI-CB (0.5 µg/mL) and (B) FAM-miR (100 nM) across diverse formulations in IEC-6 cells using flow cytometry; Figure S3: (A) The expression levels of EGFR, VCP, and PD-L1 in CT-26 and IEC-6 cells measured by Western blot analysis. (B) PDL1-targeted and intracellular distribution of FAM-miR+CB+R/SLN-CSW in CT-26 cells visualized using confocal laser scanning microscopy (CLSM); Figure S4: (A) The inhibitory concentration (IC) curve of CB on CT-26 to determine the effective concentration required for inhibition. (B) The cell viability curve utilized to calculate the combination index (CI) for the combined therapy involving miR and CB; Figure S5: Investigation of the effects of different concentrations of miR, CB, and/or R formulations on the EGFR pathway in CT-26 cells following a 24-h treatment period; Figure S6: (A) Quantification of total reactive oxygen species (ROS) levels utilizing a flow cytometer. (B) Assessment of ROS distribution, visualized through CLSM after treatment with various formulations for 24 h; Figure S7: (A) Visualization of HMGB1 via CLSM following treatment with various miR, CB, and R-loaded formulations for 24 h. (B) Assessment of intracellular ATP release using the ATP Detection Assay Kit subsequent to various treatments for 24 h; Figure S8: Examination of CD8 T cell percentages in (A) tumor and (B) spleen via flow cytometry. Quantification of CD8 T cell proportions in (C) tumor and (D) spleen; Figure S9: Assessment of T regulatory (Treg) cell proportions in (A) tumor and (B) spleen via flow cytometry. Quantification of Treg cell percentages in (C) tumor and (D) spleen; Figure S10: Evaluation of tumor-associated macrophages (TAMs) in (A) tumor and (B) spleen using flow cytometry. Quantification of TAM percentages in (C) tumor and (D) spleen; Figure S11: (A) Immunohistochemistry (IHC) examination of CD8, CD4, and Treg (FoxP3) cell distribution in spleen sections. Scale bar: 200 µm. Quantification of the stained area for (B) CD8 T cells, (C) CD4 cells, and (D) Tregs; Figure S12: Hematological parameters including red blood cell (RBC) and platelet (PLT) counts from mice following a 14-day treatment course with different formulations; Figure S13: Histopathological evaluation of biosafety in CT26-bearing mice following treatment with various formulations of CB, miR, and/or R.
