# Supplementary material for: Functional pH-Responsive Nanoparticles for Immune Reprogramming in MSS Colorectal Cancer via ER Stress-Induced Proteostasis Disruption, PD-L1-Targeting miRNA, and TLR7 Activation
Source: Pharmaceutics. 2025 Nov 20;17(11):1503. doi: 10.3390/pharmaceutics17111503 (PMC12656422; doi:10.3390/pharmaceutics17111503)
Supplement: Supplementary file 1 [file pharmaceutics-17-01503-s001.zip › pharmaceutics-3977414-supplementary.pdf]

Supplementary data

Functional pH-Responsive Nanoparticles for Immune Reprogramming in MSS Colorectal Cancer via ER Stress-Induced Proteostasis Disruption, PD-L1-Targeting miRNA, and TLR7 Activation

Yu-Li Lo [1,2,3,*,†], Hua-Ching Lin [4,5,†], Ching-Yao Li [1], Bryant Huang [1], Ching-Ping Yang [1,6], Hui-Yen Chuang [7] and Tsui-Fen Chou [8,9]

[1] Department and Institute of Pharmacology, National Yang Ming Chiao Tung University, Taipei 112, Taiwan
[2] Faculty of Pharmacy, National Yang Ming Chiao Tung University, Taipei 112, Taiwan
[3] Research Fellow, Taipei Veterans General Hospital, Taipei 112, Taiwan
[4] Division of Colorectal Surgery, Cheng-Hsin General Hospital, Taipei 112, Taiwan
[5] Department of Healthcare Information and Management, Ming Chuan University, Taoyuan 320, Taiwan
[6] Department of Medical Laboratory Science and Biotechnology, Central Taiwan University of Science and Technology, Taichung 412, Taiwan
[7] Department of Biomedical Imaging and Radiological Sciences, National Yang Ming Chiao Tung University, Taipei 112, Taiwan
[8] Division of Biology and Biological Engineering, California Institute of Technology, Pasadena, CA 91125, USA; tfchou@caltech.edu
[9] Proteome Exploration Laboratory, Beckman Institute, California Institute of Technology, Pasadena, CA 91125, USA
[*] Correspondence: yulilo@nycu.edu.tw; Tel.: +886-228-267-000 (ext. 67095)
[†] These authors contributed equally to this work.

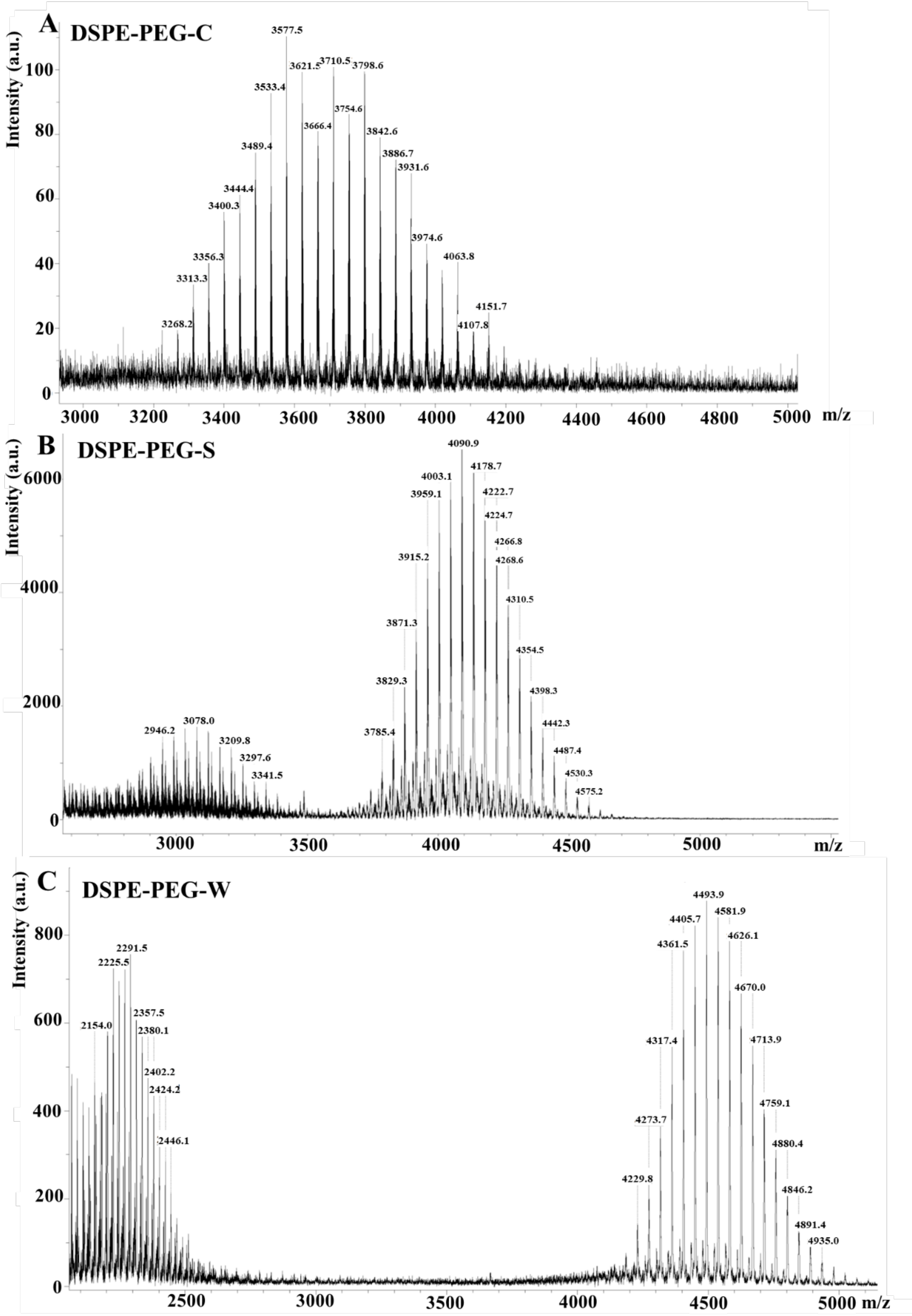

Figure S1. Mass spectra of (**A**) DSPE-PEG-C, (**B**) DSPE-PEG-S, and (**C**) DSPE-PEG-W. All data were collected via matrix-assisted laser desorption/ionization time-of-flight (MALDI-TOF) mass spectrometry.

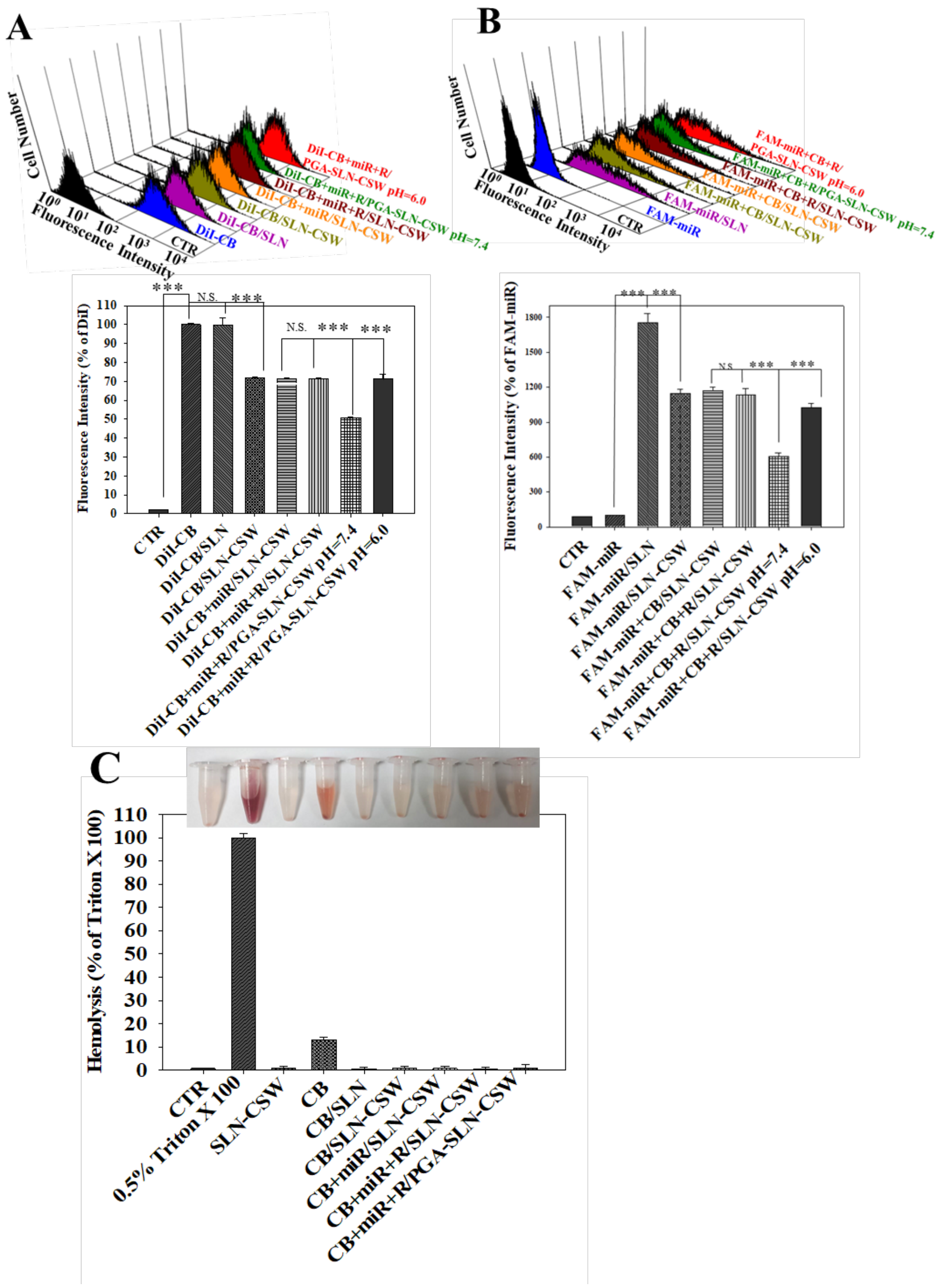

Figure S2. Quantification of intracellular fluorescence intensity of (A) DiI-CB (0.5 μg/ml) and (B) FAM-miR (100 nM) across diverse formulations in IEC-6 cells using flow cytometry. (A, B) data are presented as mean ± standard deviation (SD) of three independent trials (n = 3; N.S., no significance; ***p < 0.001 indicates statistical significance. (C) Evaluation of hemolytic effects of various formulations, with the upper panel illustrating the hemolysis, while the lower panel shows quantification of hemoglobin release from rat blood cells (RBCs).

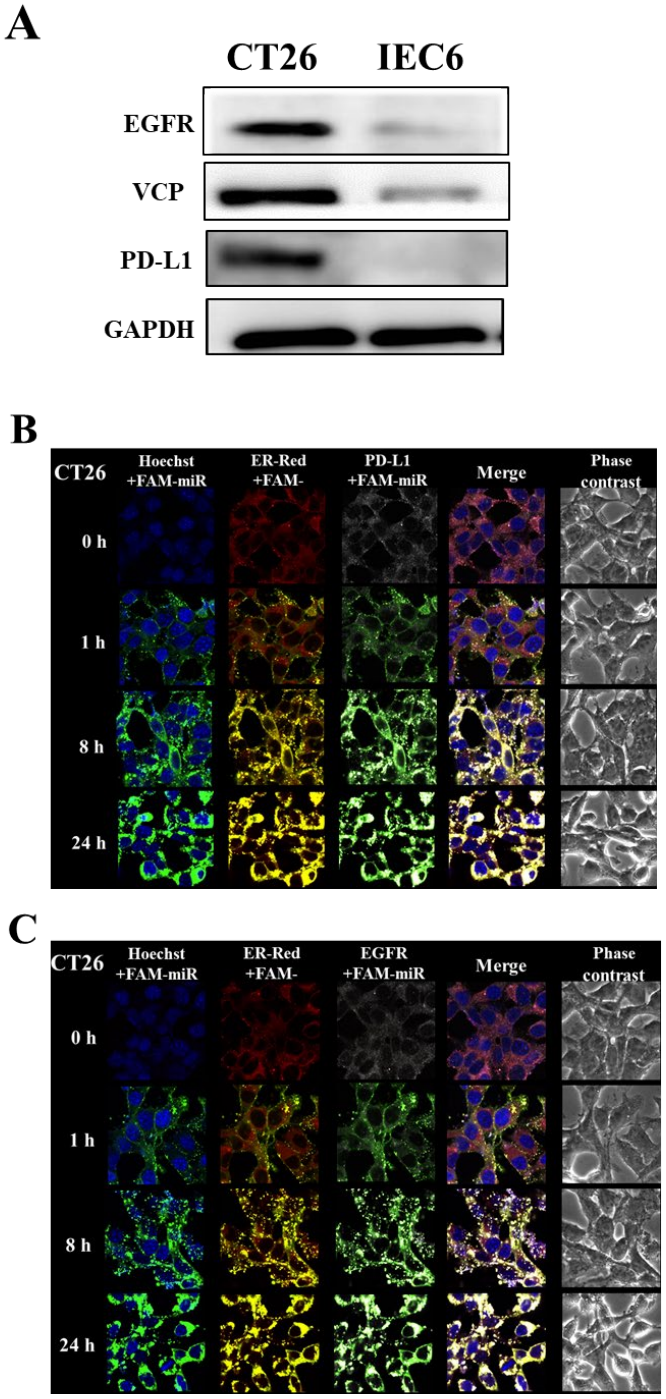

Figure S3. (**A**) The expression levels of EGFR, VCP, and PD-L1 in CT-26 and IEC-6 cells measured by Western blot analysis. (**B**) PDL1-targeted and intracellular distribution of FAM-miR+CB+R/SLN-CSW in CT-26 cells visualized using confocal laser scanning microscopy (CLSM), with blue indicating Hoechst (a nuclear dye), green representing FAM-miR, red denoting endoplasmic reticulum (ER), and gray illustrating PD-L1. Scale bar represents 20 μm. (**C**) EGFR-targeted and intracellular localization of FAM-miR+CB+R/SLN-CSW in CT-26 cells observed by CLSM, Blue: Hoechst (a nuclear dye); green: FAM-miR; red: ER; gray: EGFR. Scale bar remains at 20 μm.

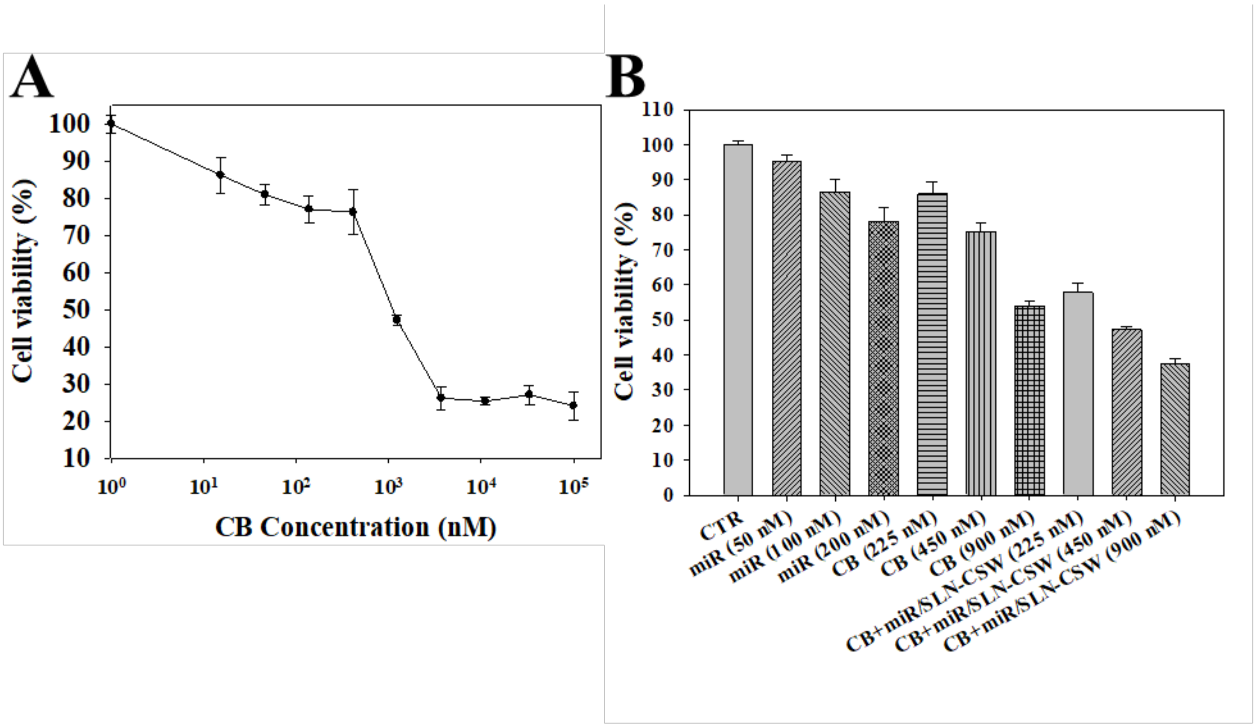

Figure S4. (**A**) The inhibitory concentration (IC) curve of CB on CT-26 to determine the effective concentration required for inhibition. (**B**) The cell viability curve utilized to calculate the combination index (CI) for the combined therapy involving miR and CB.

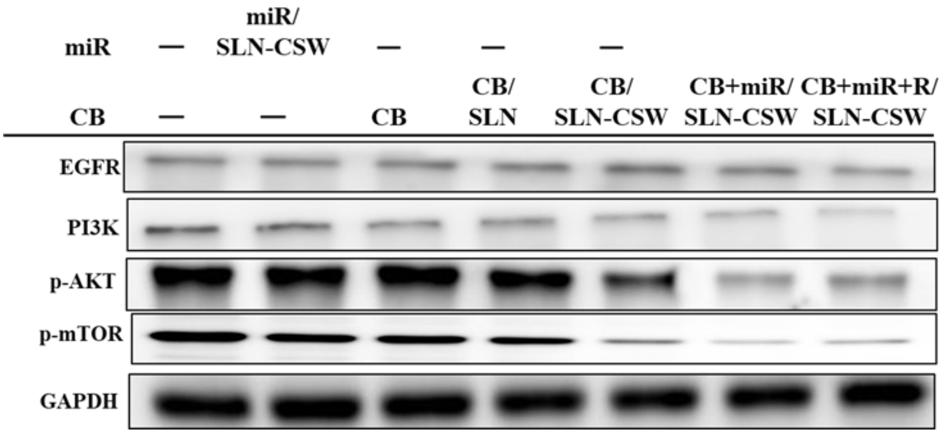

Figure S5. Investigation of the effects of different concentrations of miR, CB, and/or R formulations on the EGFR pathway in CT-26 cells following a 24-h treatment period.

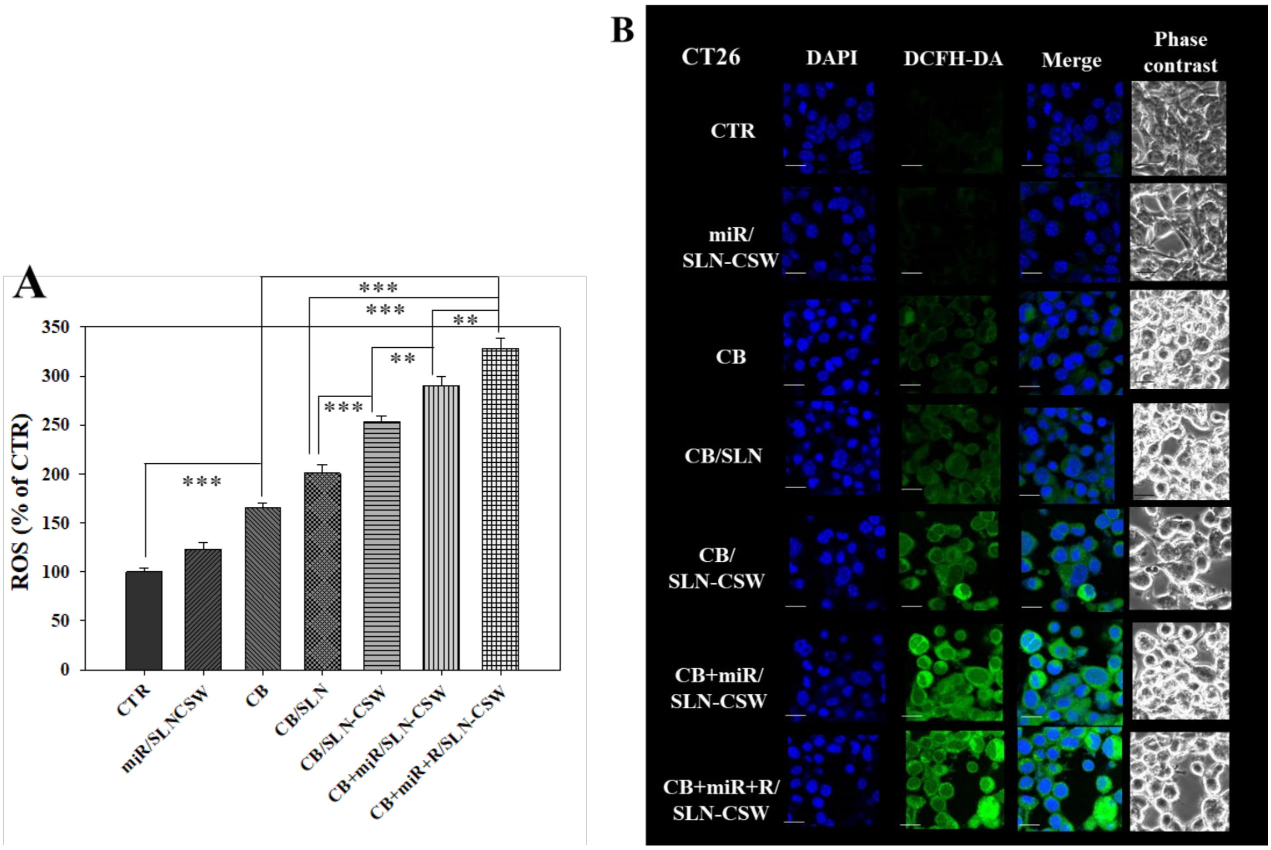

Figure S6. (A) Quantification of total reactive oxygen species (ROS) levels, determined by staining with the fluorescent probe 2',7'-dichlorodihydrofluorescein diacetate (DCFH-DA), utilizing a flow cytometer (*statistical significance at $^{**}p < 0.01$ and $^{***}p < 0.001$). (B) Assessment of ROS distribution, visualized through CLSM after treatment with various formulations for 24 h. Blue represents DAPI (a nuclear dye), while green indicates ROS. Scale Bar: 20 μm.

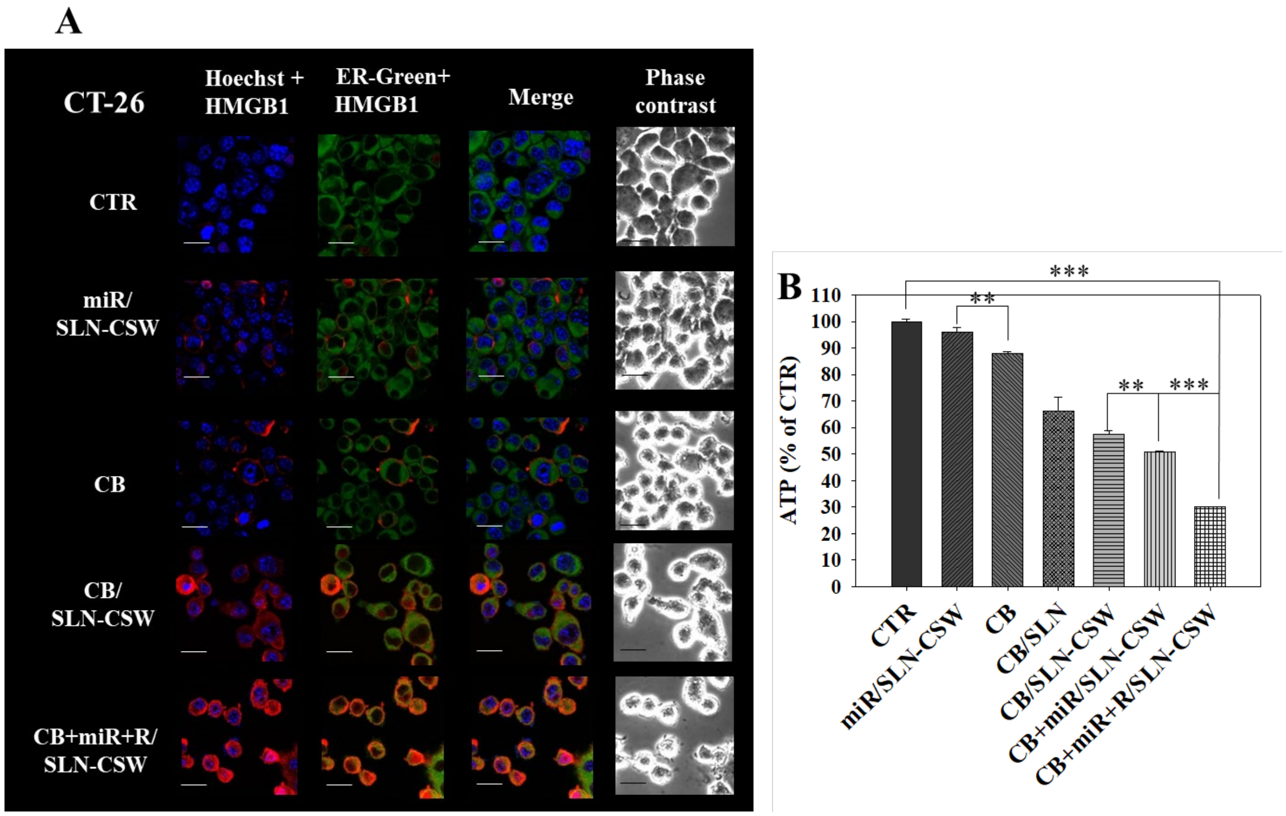

Figure S7. (**A**) Visualization of HMGB1 via CLSM following treatment with various miR, CB, and R-loaded formulations for 24 h. Hoechst (blue) stains nuclei, ER (green) indicates endoplasmic reticulum, and HMGB1 (red) depicts the target protein. Scale Bar: 20 µm. (**B**) Assessment of intracellular ATP release using the ATP Detection Assay Kit subsequent to various treatments for 24 h (*denotes statistical significance at ** $p < 0.01$ and *** $p < 0.001$).

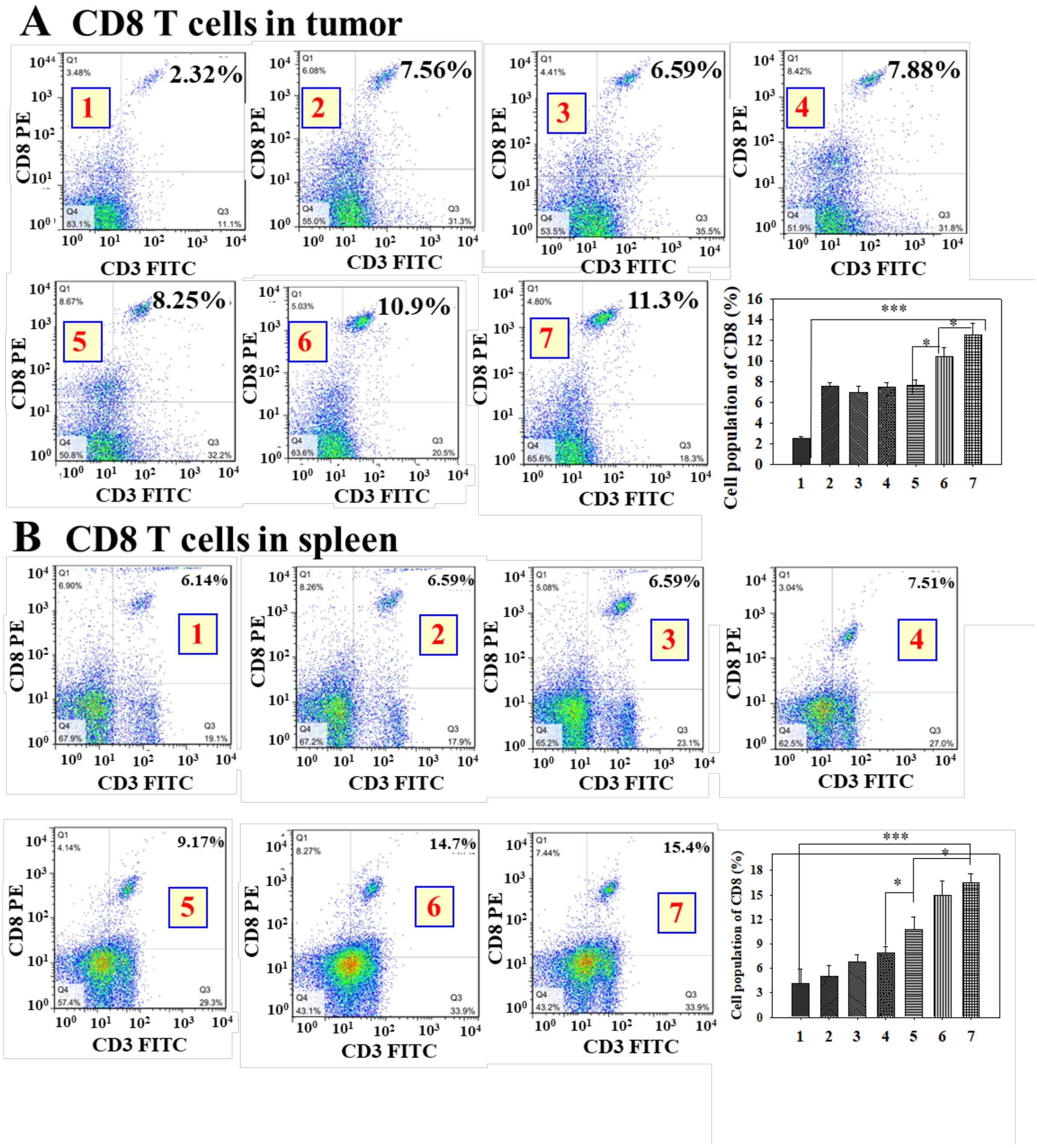

Figure S8. Examination of CD8 T cell percentages in (A) tumor and (B) spleen via flow cytometry. Quantification of CD8 T cell proportions in (C) tumor and (D) spleen (N.S., no significance, *statistical significance at $*p < 0.05$ and $***p < 0.001$).

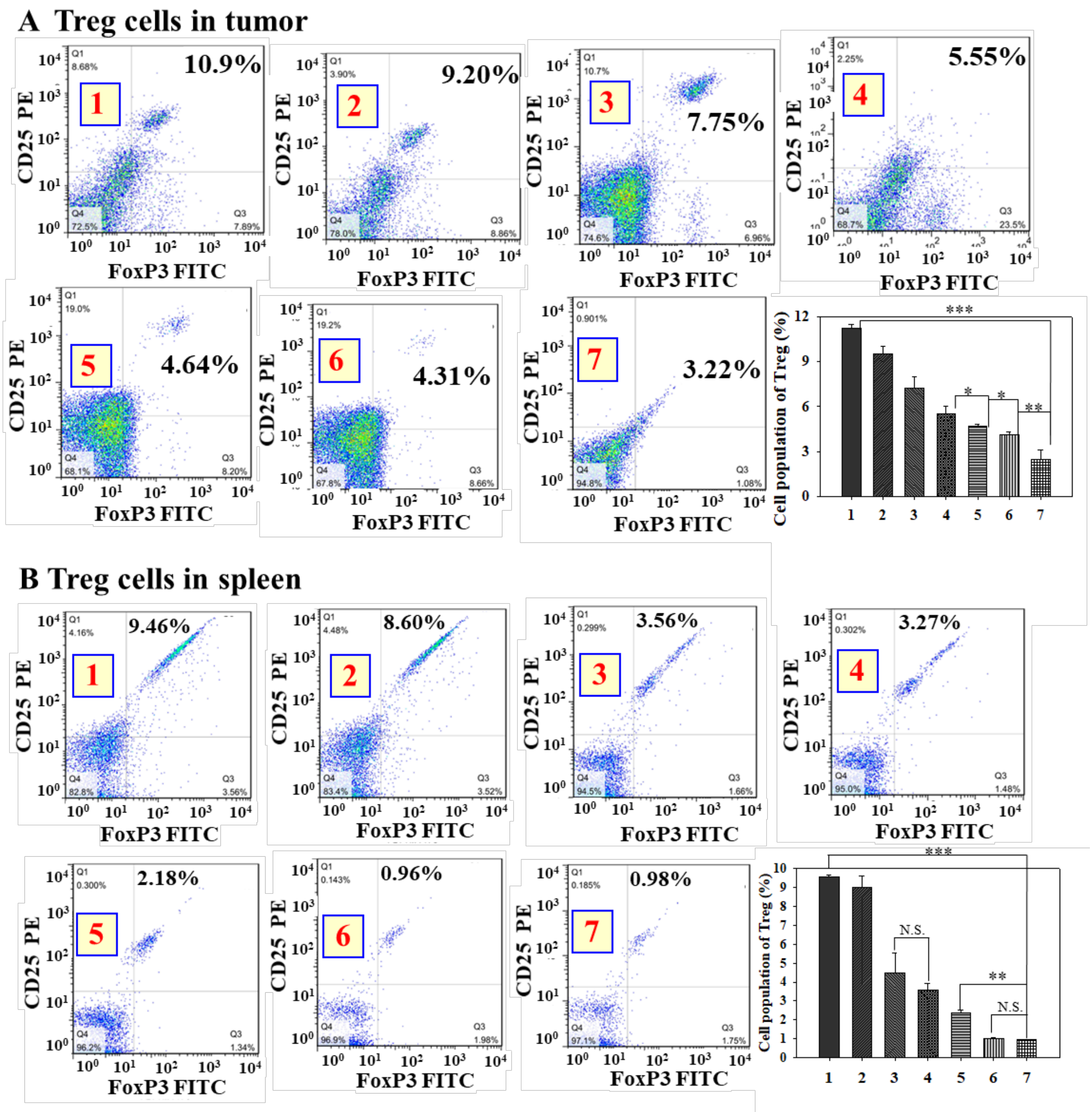

Figure S9. Assessment of T regulatory (Treg) cell proportions in (A) tumor and (B) spleen via flow cytometry. Quantification of Treg cell percentages in (C) tumor and (D) spleen (N.S., no significance, *statistical significance at *$p < 0.05$, **$p < 0.01$, and ***$p < 0.001$).

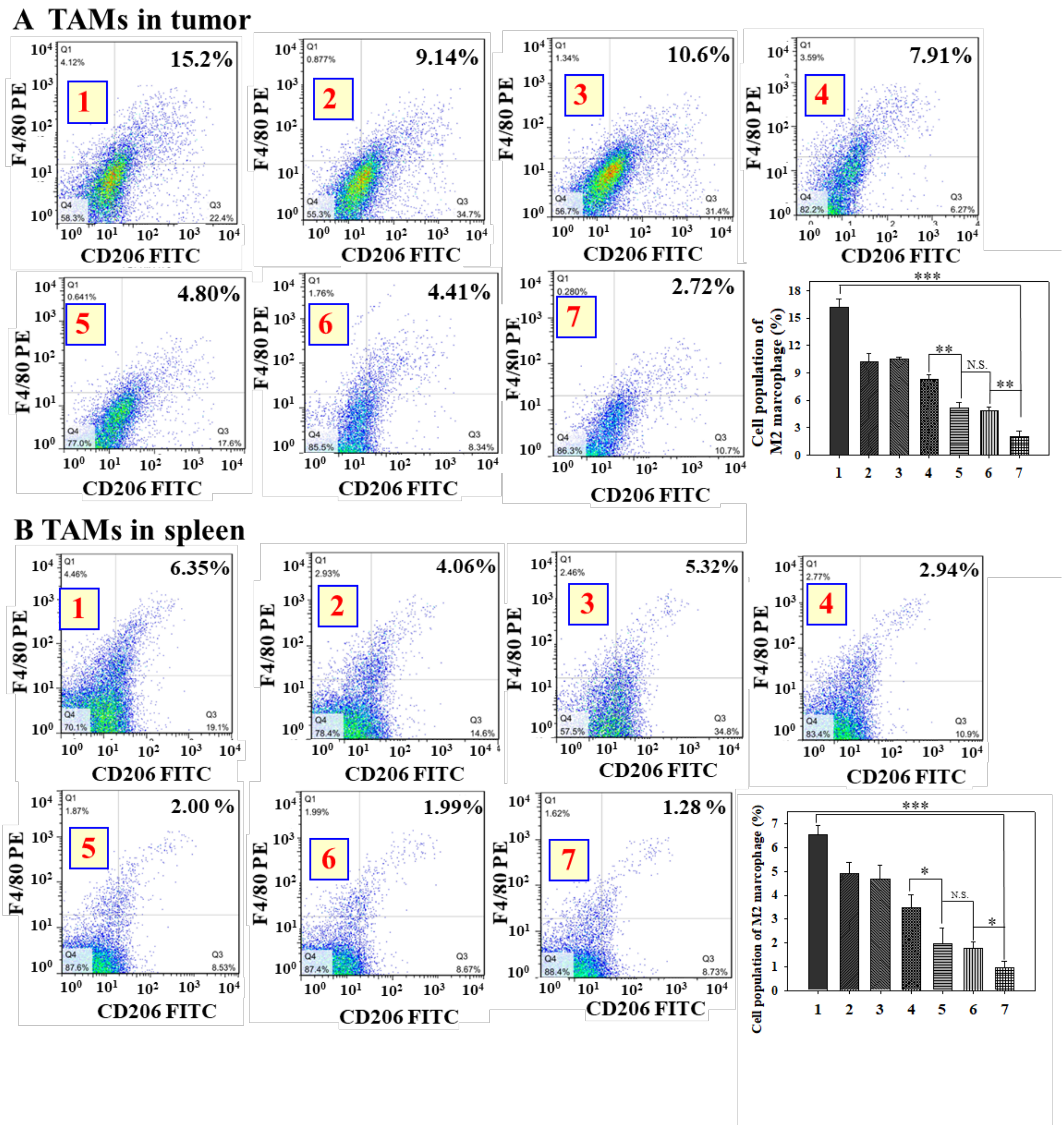

Figure S10. Evaluation of tumor-associated macrophages (TAMs) in (A) tumor and (B) spleen using flow cytometry. Quantification of TAM percentages in (C) tumor and (D) spleen (N.S., no significance, *statistical significance at *$p < 0.05$, **$p < 0.01$, and ***$p < 0.001$).

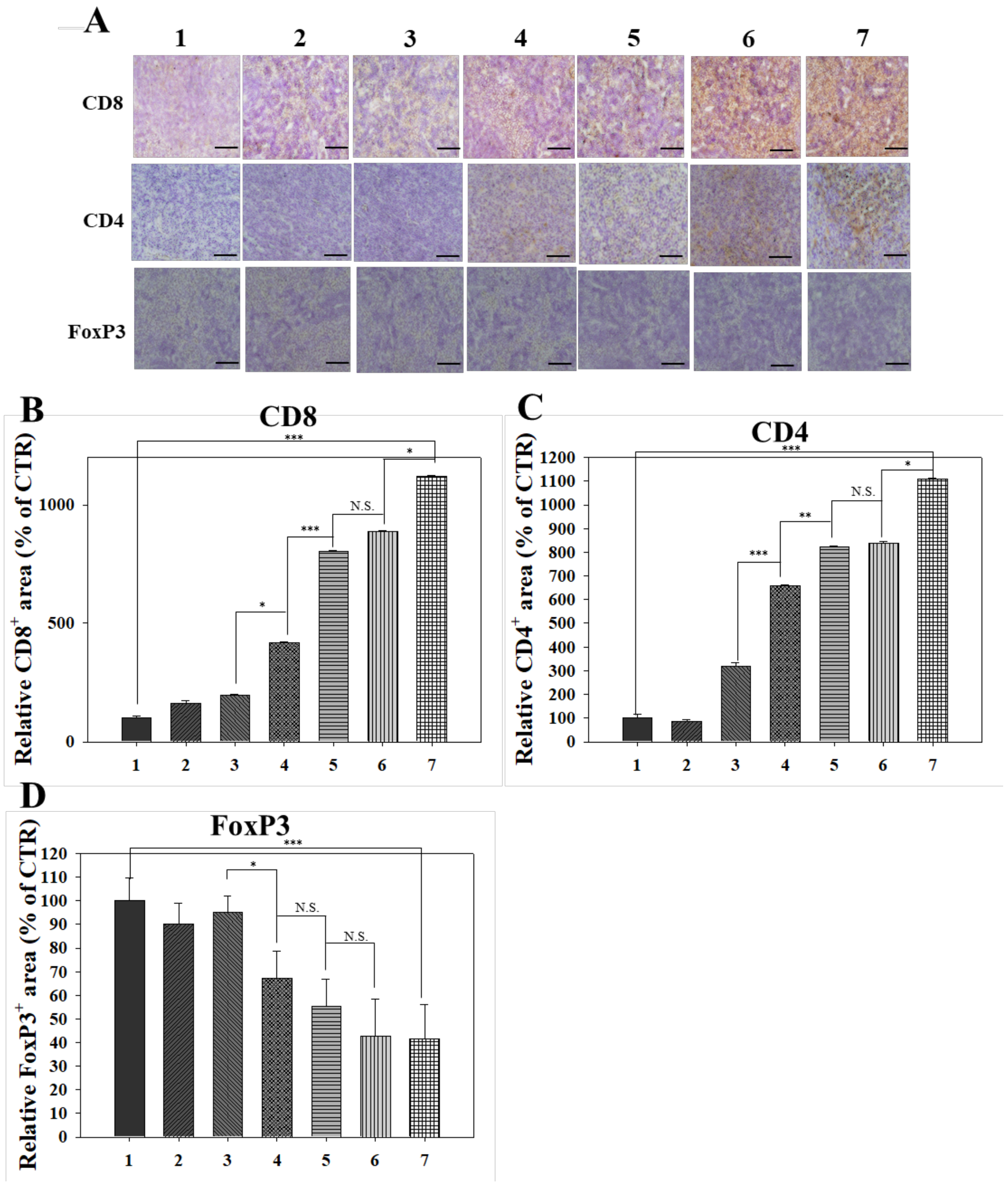

Figure S11. (**A**) Immunohistochemistry (IHC) examination of CD8, CD4, and Treg (FoxP3) cell distribution in spleen sections. Scale bar: 200 μm. Quantification of the stained area for (**B**) CD8 T cells, (**C**) CD4 cells, and (**D**) Tregs (N.S., no significance, *statistical significance at $^{*}p < 0.05$, $^{**}p < 0.01$, and $^{***}p < 0.001$).

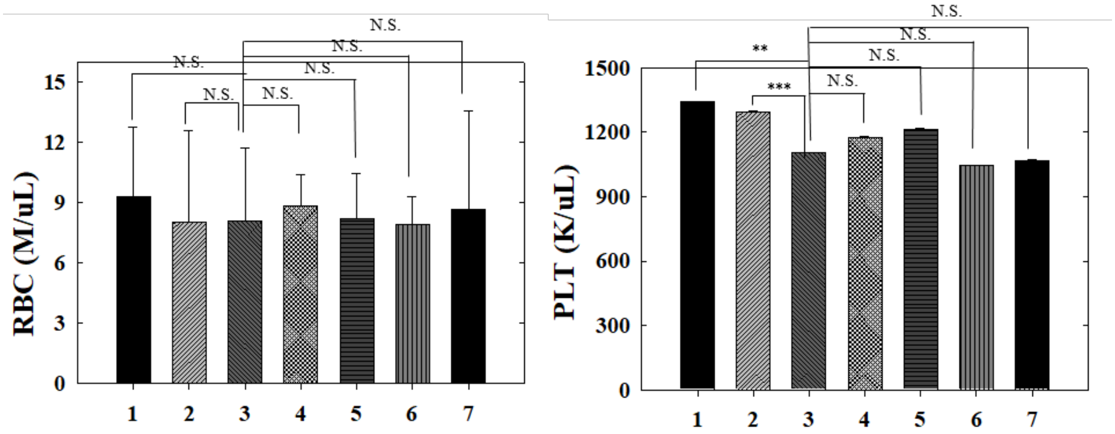

Figure S12. Hematological parameters including red blood cell (RBC) and platelet (PLT) counts from mice following a 14-day treatment course with different formulations (N.S., no significance, *statistical significance at ***p* < 0.01 and ****p* < 0.001).

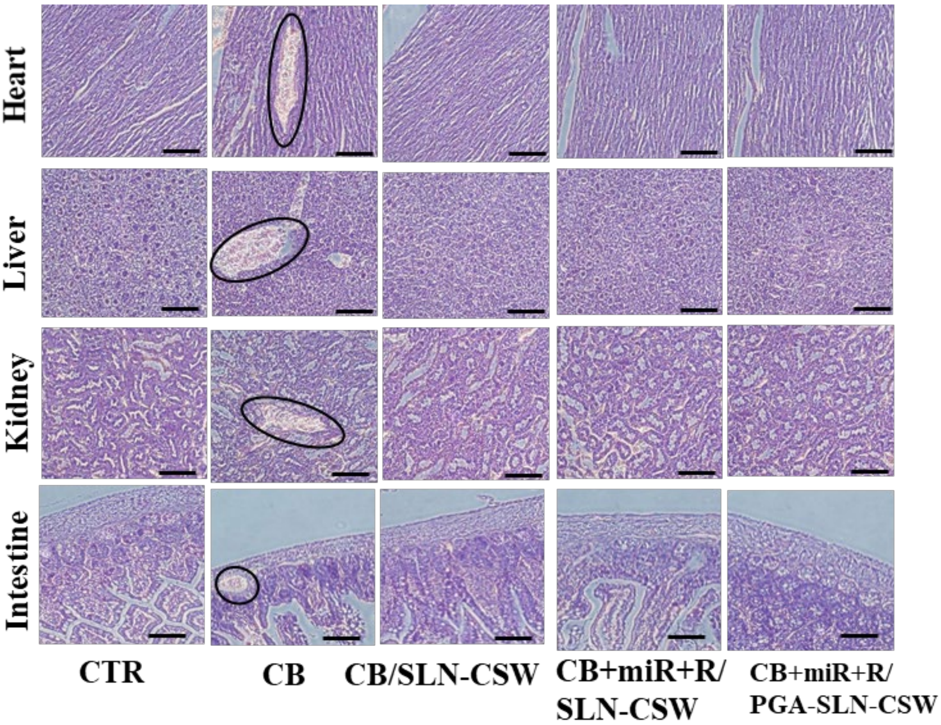

Figure S13. Histopathological evaluation of biosafety in CT26-bearing mice following treatment with various formulations of CB, miR, and/or R. Representative H&E-stained sections of the kidney, liver, heart, and intestine are shown. Black ovals highlight regions of abnormal cellular architecture, infiltration, or structural disruption. Scale bar: 200 μm.
